# AML risk stratification models utilizing ELN-2017 guidelines and additional prognostic factors: a SWOG report

**DOI:** 10.1186/s40364-020-00208-1

**Published:** 2020-08-12

**Authors:** Era L. Pogosova-Agadjanyan, Anna Moseley, Megan Othus, Frederick R. Appelbaum, Thomas R. Chauncey, I-Ming L. Chen, Harry P. Erba, John E. Godwin, Isaac C. Jenkins, Min Fang, Mike Huynh, Kenneth J. Kopecky, Alan F. List, Jasmine Naru, Jerald P. Radich, Emily Stevens, Brooke E. Willborg, Cheryl L. Willman, Brent L. Wood, Qing Zhang, Soheil Meshinchi, Derek L. Stirewalt

**Affiliations:** 1 Clinical Research Division, Fred Hutch, 1100 Fairview Ave N, D5-112, Seattle, WA 98109 USA; 2 SWOG Statistical Center, Fred Hutch, Seattle, WA USA; 3 Departments of Oncology and Hematology, University of Washington, Seattle, WA USA; 4 VA Puget Sound Health Care System, Seattle, WA USA; 5 Department of Pathology, University of New Mexico, UNM Comprehensive Cancer Center, Albuquerque, NM USA; 6 Duke Cancer Institute, Durham, NC USA; 7 Providence Cancer Center, Earle A. Chiles Research Institute, Portland, OR USA; 8 Clinical Biostatistics, Fred Hutch, Seattle, WA USA; 9 Departments of Laboratory Medicine and Pathology, University of Washington, Seattle, WA USA; 10 Malignant Hematology, H. Lee Moffitt Cancer Center & Research Institute, Tampa, FL USA; 11 Bioinformatics Shared Resource, Fred Hutch, Seattle, WA USA; 12 Department of Pediatrics, University of Washington, Seattle, WA USA

**Keywords:** AML, Acute myeloid leukemia, Prognostic factors, Mathematical modeling, Elderly, Biomarkers, European LeukemiaNet guidelines, ELN, Model development and validation

## Abstract

**Background:**

The recently updated European LeukemiaNet risk stratification guidelines combine cytogenetic abnormalities and genetic mutations to provide the means to triage patients with acute myeloid leukemia for optimal therapies. Despite the identification of many prognostic factors, relatively few have made their way into clinical practice.

**Methods:**

In order to assess and improve the performance of the European LeukemiaNet guidelines, we developed novel prognostic models using the biomarkers from the guidelines, age, performance status and select transcript biomarkers. The models were developed separately for mononuclear cells and viable leukemic blasts from previously untreated acute myeloid leukemia patients (discovery cohort, *N* = 185) who received intensive chemotherapy. Models were validated in an independent set of similarly treated patients (validation cohort, *N* = 166).

**Results:**

Models using European LeukemiaNet guidelines were significantly associated with clinical outcomes and, therefore, utilized as a baseline for comparisons. Models incorporating age and expression of select transcripts with biomarkers from European LeukemiaNet guidelines demonstrated higher area under the curve and C-statistics but did not show a substantial improvement in performance in the validation cohort. Subset analyses demonstrated that models using only the European LeukemiaNet guidelines were a better fit for younger patients (age < 55) than for older patients. Models integrating age and European LeukemiaNet guidelines visually showed more separation between risk groups in older patients. Models excluding results for *ASXL1*, *CEBPA*, *RUNX1* and *TP53*, demonstrated that these mutations provide a limited overall contribution to risk stratification across the entire population, given the low frequency of mutations and confounding risk factors.

**Conclusions:**

While European LeukemiaNet guidelines remain a critical tool for triaging patients with acute myeloid leukemia, the findings illustrate the need for additional prognostic factors, including age, to improve risk stratification.

## Background

The recently revised European LeukemiaNet (ELN-2017) recommendations for diagnosis and management of adult patients with acute myeloid leukemia (AML) are broadly accepted by physicians as a gold standard and provide guidelines to stratify patients into three outcome groups: favorable, intermediate, and adverse based on cytogenetics and mutation status of *ASXL1*, *CEBPA*, *FLT3*, *NPM1*, *RUNX1*, and *TP53* [1]. This stratification scheme provides a simple, yet powerful means to triage patients for appropriate therapies. Two previous studies have validated the performance of ELN-2017 guidelines in patients ≤65 years old with AML [2, 3] and one study has evaluated the use of non-coding RNA expression to improve the prognostic significance of the ELN-2017 risk classification [4]. However, the majority of AML patients are older than the previously studied patients [5], and clinical prognostic factors such as age and performance status (PS) were not examined in the previous studies, nor are these factors included in the ELN-2017 guidelines [1, 6–8]. Similarly, prognostic guidelines, including ELN-2017, do not provide recommendations or guidance about the optimal material for clinical testing. Prognostic biomarkers have generally been identified, optimized, and validated using cryopreserved samples comprised of heterogeneous populations of mononuclear cells (MNCs). The inter-sample variability in the percentage and viability of leukemic blasts may impact continuous biomarkers like *FLT3*-ITD allelic ratio (AR) or transcript expression [6]. Thus, studies are needed to determine if examining a more homogenous population of malignant cells may improve the precision of risk stratification guidelines, and these studies, including those examining the current ELN-2017 guidelines, need to be extended to older patients [5, 9–12].

Therefore, we developed novel prognostic models using ELN-2017 risk stratification guidelines (ELN_2017_), clinical factors such as age and PS, and expression of select transcripts reported to be associated with prognosis [6, 13–27]. The models provided continuous risk scores that were used to define risk stratification thresholds. The models were developed separately for unsorted MNCs and highly enriched viable leukemic blasts (VLBs) to examine the potential prognostic benefit of testing a more homogenous population of malignant cells, representing the largest systematic evaluation of paired MNCs and VLBs from patients with AML to date. These risk models were then validated in an independent population of patients. Given that clinical assays for *ASXL1*, *CEBPA*, *RUNX1* and *TP53* are not available at every institution, we also examined the performance of models without the mutation status of these four genes (ELN_2017-MOD_). The results demonstrated the utility of the ELN-2017 guidelines for younger patients with AML and caution for applying the same risk strata to older patients. Integration of the selected expression biomarkers into models did not markedly improve the model performance. In addition, the findings highlight the need for new prognostic biomarkers and risk stratification approaches for older adults with AML.

## Methods

### Patient materials

A review of SWOG Cancer Research Network leukemia repository inventory identified 383 out of 1042 previously untreated AML patients 1) with pretreatment bone marrow or peripheral blood samples containing ≥3 cryopreserved vials and 2) who were enrolled onto trials to receive cytarabine- and daunorubicin-based induction chemotherapy and consolidation with curative intent. These patients were enrolled onto protocols SWOG-9031, SWOG-9333, S0106 and S0112 and treated as previously described [28–31]. The included patients were assigned into two cohorts by simple randomization: a discovery cohort (*n* = 190) and a validation cohort (*n* = 193) by the SWOG Statistical Center [32]. Specimen handling and cryopreservation were consistent across the studies per previously described standard operating procedures [6]. All participants provided written informed consent to participate in correlative research in compliance with the Declaration of Helsinki, and all studies were conducted with the approval of Fred Hutch Institutional Review Board.

### Thawing, FACS preparation, analyses, and nucleic acid extraction

Cryopreserved samples were thawed as previously described [6]. A portion of unsorted MNCs was lysed, while the remainder of the sample underwent fluorescence-activated cell sorting (FACS) to isolate VLBs using forward-by-side scatter, DAPI staining and fluorescently-labeled antibodies to CD45, CD34 and CD117 as previously described [6]. RNA and DNA from unsorted MNCs and VLBs were extracted and quantified as previously described [6]. Of the randomly selected samples, 185/190 (97%) and 166/193 (86%) yielded sufficient materials in each cohort for downstream analyses.

### Identification of genomic mutation

Internal tandem duplication in *FLT3* (*FLT3*-ITD) and mutations in *NPM1* were assayed via fragment analyses as previously described [7, 33, 34]. If the wild type *FLT3* was not detected in a sample with an ITD, an allelic ratio (AR) of 20 was assigned [35]. MNCs from the discovery cohort were sequenced for mutations in *ASXL1*, *RUNX1* and *TP53* using Wafergen technology by the British Columbia Cancer Agency (BCCA) per standard protocols (Additional File, Tables S1A and B). VLBs from the discovery and validation cohorts were sequenced using TruSight™ Myeloid Sequencing Panel (Illumina) as previously described [36]. Neither BCCA nor TruSight™ platforms provided optimal coverage for *CEBPA*, therefore, targeted MiSeq *CEBPA* assays were developed (Additional File, Table S1C). Paired-end short reads were first aligned to the human genome GRCh37/hg19 reference assembly using Burrows-Wheeler Aligner, BWA, v0.7.12 [37]. The resulting alignment data were further processed based on the best practice of Genome Analysis Toolkit (GATK, v3.5 https://www.broadinstitute.org/gatk/). The overview of sequence alignment statistics was computed for each sample using Samtools (v1.0 http://samtools.sourceforge.net), and the sequence coverage was computed for each sample using GATK DepthOfCoverage. Variants were called per sample using GATK HaplotypeCaller in GVCF mode, then jointly as a cohort using GenotypeGVCFs. The resulting collection of variants, in the form of a VCF file, were annotated using Annovar, version 2016Feb01 [38]. The following exclusion filters were applied: synonymous variants, low quality (Qual score < 150), variant read depth < 65 [39], variant allele frequency (VAF) < 10%, and non-exonic loci outside of splice sites. For in-frame missense amino acid substitutions, additional filters excluded changes with a FATHMM_score <  0.7 [40] and/or if ExAC_ALL score was > 0.0001, unless the missense amino acid change was defined as likely pathogenic or pathogenic by ClinVar and described as somatic in the COSMIC databases (https://cancer.sanger.ac.uk) [41]. Sequencing depth for each read loci were calculated to determine average percent coverage. For quality control (QC), loci with > 20% of samples displaying < 65 read coverage were removed from downstream analyses. Three loci failed QC, but the excluded loci displayed a very low abundance of previously reported somatic mutations confirmed to be present in hematopoietic malignancies in Cosmic Database (Additional File, Table S2).

### Expression of transcript biomarkers

Taqman gene expression assays for *BAALC*, *CEBPA*, *CCNA1*, *CD34*, *ERG1*, *EVI1*, *FLT3*, *GATA2*, *IL3RA*, *JAG1*, *KIT*, *MN1*, *RUNX1*, and *WT1* were used to quantify gene expression as previously described [6, 22]. The fold change (FC) for each transcript was computed using the comparative Ct method with Beta-glucuronidase (*GUSB*) normalization to pooled non-malignant bone marrow calibrator, except for *WT1* FC, which used LAMA-84 cell line as a calibrator [6]. The FC was censored at maximum cycle threshold of 45 for samples without evidence of expression by qRT/PCR. Transcript expression was examined in experimental duplicates, with the geometric mean of the duplicates used for downstream analyses. If either of the replicates was censored, their mean was also marked as censored. If a duplicate was not available, we used the FC and censoring of that single expression value. Censored values were assigned the minimum expression observed for that gene, divided by the square root of two [42].

### Statistical analyses

Cytogenetic and mutation risk classification was based on the ELN-2017 guidelines [1]. Complete remission (CR) required the following: > 20% marrow cellularity with maturation of all cell lines, < 5% blasts, no Auer rods, absolute neutrophil count (ANC) ≥1500/μL, platelets > 100,000/μL, no peripheral blasts, and no extramedullary disease. Study S0106 required ANC ≥1000/μL and did not have any marrow cellularity or peripheral blasts requirements. Overall survival (OS) was measured from the date of study registration to the date of death by any cause, with patients last known to be alive censored at the date of last contact. Relapse-free survival (RFS) was measured from date of CR to date of death or relapse, with patients last known to be alive and without report of relapse censored at the date of last contact. Transplant data were not collected on these trials and, thus, are not available for incorporation into the modeling algorithms. Disease characteristics, patient demographics, and clinical responses were compared between the pool of patients who were selected to be analyzed herein (*N* = 351) versus patients enrolled on the four trials who were not analyzed (*N* = 691) using Chi-squared, Fisher’s exact, or Wilcoxon rank-sum tests as appropriate. The same analyses were used to compare the discovery (*N* = 185) and validation (*N* = 166) cohorts.

Differences in mutation status, gene expression, and *FLT3*-ITD AR in paired MNC and VLB samples were assessed using McNemar’s test or the Wilcoxon signed rank test, as appropriate. OS and RFS were estimated using the Kaplan-Meier method and compared across groups using log-rank tests. RFS and OS models used Cox proportional hazards regression; CR models used logistic regression. Model building in the discovery cohort was composed of the following steps, done separately for each outcome and for each type of material (unsorted MNCs and VLBs). 1) Univariate models were fit for each of the following baseline variables: age (quantitative), performance status (0–1 vs. 2–3), AML onset (secondary vs de novo), clinical trial, immunophenotype (IP) and ELN-2017 risk group. 2) Multivariable models were fit with covariates with *p*-value < 0.10 from step 1 for each of the expression variables. These adjusted expression *p*-values were ranked, and the 5 expression variables with the smallest p-values were selected for additional modeling. If ELN-2017 risk group or IP were included in the multivariable models, interactions with expression variables were also evaluated, and interactions with p-values less than 0.15 were selected for additional modeling; if more than 5 interactions had p-values less than 0.15, the 5 with the smallest p-values were selected for additional modeling. 3) A multivariable model including selected baseline variables, selected expression variables, and selected interaction variables was built using backwards selection based on the Aikike Information Criterion (AIC). Area under the Receiver Operating Characteristic curve (AUC) and C-statistics were estimating using 5-fold cross validation of the entire (3-step) model building process. The locked parameter values from step 3 were fit to the validation cohort and AUC and C-statistics were calculated. We note that AUC and C-statistic values of 0.50 indicate prediction equivalent to a coin flip (random prediction), and values of 1.00 indicate perfect prediction. Analyses were performed using SAS version 9.4 (SAS Institute, Cary NC) and R version 3.4.3 [43].

## Results

### Characteristics of patient population

Patients who were included in this study displayed higher WBC, blast percentage, and ANC (*P* <  0.0001 for all) compared to patients enrolled on these trials who were not included in this study. In addition, there was a significant difference in cytogenetic profiles (*P* = 0.0031), FAB class (*P* < 0.0001), and proportions across clinical trials (*P* = 0.0129, Additional File, Table S3). These differences between included and not included patients likely reflect reported biases for patients within repositories having a higher burden of disease at diagnosis and depletion of specimens from older trials [6]. The differences between trial representation likely reflect the higher abundance of samples from the more recent trials. However, there were no significant differences between the included and not included patients with respect to CR rates (60% vs. 58% *P* = 0.52), RFS (5-year RFS 32% vs. 33%; P = 0.52) or OS (5-year OS 30% vs. 32%; *P* = 0.62, Additional File, Table S3 and Fig. S1). Comparing the discovery and validation patients, the two cohorts displayed some differences in clinical characteristics despite randomization (e.g., WBC, *P* = 0.0188; cytogenetics, *P* = 0.0296; cytogenetics risk group, *P* = 0.028 and distribution across clinical trials, *P* = 0.0209; Additional File, Table S4), however there were no significant differences in clinical outcomes between the discovery and validation cohorts (CR 57% vs. 63% *P* = 0.31; 5-yr RFS 30% vs. 34%; *P* = 0.54; or 5-yr OS 30% vs. 31%; *P* = 0.82, Additional File, Table S4 and Fig. S2).

### Characterization of mutations and transcript expression

Mutation analyses focused on genes utilized for ELN-2017 risk stratification. *FLT3*-ITD and *NPM1* mutations were examined in all specimens with available material (i.e., MNCs and VLBs). There was 100% concordance for *NPM1* mutations in MNCs and VLBs. One *FLT3*-ITD was observed in the MNCs but not VLBs (99.7% concordant). *FLT3*-ITD and *NPM1* mutations were detected in 109 (31%) and 125 (36%) patients, respectively. The distribution and mutation frequencies of *NPM1* and *FLT3*-ITD, as well as *FLT3*-ITD AR, were not significantly different between discovery and validation cohorts in either population of cells (Additional File, Table S5 and Fig. S3). Excluding the patient with discordant *FLT3*-ITD results, *FLT3*-ITD AR was significantly higher in VLBs than the MNCs (AR ranges 0.03–20 and 0.04–13.2, respectively, *P* < 0.0001). Given that the ELN-2017 guidelines utilize *FLT3*-ITD AR of 0.5 for risk stratification, we examined the impact that testing the *FLT3*-ITD AR in VLBs had on ELN-2017 classification. In the MNCs, percentages of patients with low and high *FLT3*-ITD ARs were 34 and 66%, respectively, while percentages for low and high AR in VLBs were 23 and 77%. Examining *FLT3* in VLBs resulted in a different AR classification for 19 patients, with 15 patients changing from low AR in MNCs to high AR in VLBs and 4 patients changing from high AR in MNCs to low AR in VLBs.

*ASXL1*, *CEBPA*, *RUNX1,* and *TP53* mutations were examined in both MNC and VLB populations for the discovery cohort. Similar to the results for *NPM1* and *FLT3*, there was a 99.4% concordance in mutations between MNCs and VLBs, with only one patient displaying a discrepancy for an *ASXL1* mutation. Therefore, mutation analyses for *ASXL1*, *CEBPA*, *RUNX1*, and *TP53* were examined in only VLBs for the validation cohort. Overall, the frequencies of mutations in the examined patients were as follows: *ASXL1* (*N* = 35, 10%), *CEBPA* (*N* = 20, 6%), *RUNX1* (*N* = 40, 11%), and *TP53* (*N* = 26, 7%). The frequency of *ASXL1* mutations was modestly higher in the discovery cohort (13% discovery vs. 7% validation, *P* = 0.044); other mutations displayed similar frequencies in both groups of patients (Additional File, Table S5 and Fig. S3).

Building upon the results examining transcript biomarkers in the discovery cohort [6], analyses examined transcript expression as a continuous variable for 13 genes, which had been previously reported to be potential prognostic biomarkers: *BAALC*, *CCNA1*, *CEBPA*, *ERG1*, *EVI1*, *FLT3*, *GATA2*, *IL3RA*, *JAG1*, *KIT*, *MN1*, *RUNX1*, and *WT1* [13–27]. In the case of *EVI1*, transcript expression was not detectable and thus censored in 69% of VLBs and 70% of MNCs. Given the dichotomous nature of *EVI1* expression, we also examined the prognostic significance of *EVI1* expression as a binary variable (expressed vs. not expressed). In the discovery cohort, univariate analyses showed a significant increase in expression in VLBs relative to MNCs for *BAALC* (*P* < 0.0001), *CCNA1* (*P* = 0.005), *ERG1* (P < 0.0001), *EVI1* (*P* = 0.001), *FLT3* (*P* = 0.024), *MN1* (P < 0.0001), *RUNX1* (P = 0.001) and *WT1* (P < 0.0001), while none of the transcripts were expressed at significantly lower levels in VLBs than MNCs (Additional File, Table S6).

### Prognostic significance of biomarkers in univariate analyses

Univariate analyses examined the prognostic significance of *FLT3*-ITD AR, *NPM1* mutation, and transcript expression in MNCs and VLBs in the discovery cohort. Increasing *FLT3*-ITD AR in MNCs was associated with worse OS (Table 1). *NPM1* mutations were not associated with clinical outcome in univariate analyses (Table 1). The prognostic significance for some transcripts varied depending upon tested cell type (Table 1, Additional File, Table S7). Overall, increased expression of *CCNA1*, *ERG1*, *EVI1*, *FLT3*, *IL3RA, KIT* and *MN1* was significantly associated with adverse risk for one or more clinical outcomes in one or both cell populations (Table 1), while expression of *BAALC*, *CEBPA*, *GATA2*, *JAG1*, *RUNX1* and *WT1* were not significantly associated with clinical outcomes in either MNCs or VLBs (Additional File, Table S7). Univariate analyses also evaluated the prognostic significance of age, cytogenetics, PS, secondary AML status, and ELN risk groups in the discovery cohort. As expected, increasing age, adverse cytogenetics, poor PS, and secondary AML status were significantly associated with poor clinical outcomes (Table 2). Favorable ELN-2017 risk was significantly associated with improved CR, whether examining MNCs or VLBs (OR = 3.11, *P* = 0.024 and OR = 3.69, *P* = 0.014, respectively), while adverse and unknown ELN-2017 risks were not significantly associated with CR (Table 2). Favorable ELN-2017 risk was also significantly associated with improved OS in VLBs (MNCs, HR = 0.58, *P* = 0.060 and VLBs, HR = 0.38, *P* = 0.001, Table 2). Adverse ELN-2017 risk was associated with reduced OS in MNCs (HR = 1.66, *P* = 0.050) but not in VLBs (HR = 1.10, *P* = 0.720). In keeping with the CR and OS analyses, favorable ELN-2017 was significantly associated with improved RFS in both MNCs and VLBs (HR = 0.47, *P* = 0.027 and HR = 0.37, *P* = 0.008, respectively, Table 2).

**Table 1:** Genomic and transcript biomarkers significant in the discovery cohort

**Variable**		**Cell Population**			
**Biomarker**	**Value**	**MNCs**		**Blasts**	
**Complete Response, CR**		**OR (95% CI)**	**P**	**OR (95% CI)**	**P**
*FLT3*-ITD AR	Continuous	0.61 (0.27–1.36)	0.229	0.92 (0.75–1.14)	0.446
	≥ 0.5 vs. < 0.5	1.15 (0.38–3.51)	0.801	0.83 (0.24–2.84)	0.770
*NPM1*	Mutated vs. not	1.38 (0.74–2.59)	0.310	1.43 (0.76–2.72)	0.270
*ERG1*	Cont.	0.94 (0.88–1.00)	0.042	0.94 (0.90–0.99)	0.018
	IQR	0.73 (0.54–0.99)		0.63 (0.43–0.93)	
*EVI1*	Binary	0.46 (0.24–0.89)	0.020	0.38 (0.19–0.76)	0.006
	Cont.	0.99 (0.98–1.00)	0.196	0.99 (0.98–1.01)	0.257
	IQR	1.00 (1.00–1.00)		1.00 (1.00–1.00)	
*KIT*	Cont.	0.96 (0.91–1.01)	0.145	0.94 (0.88–0.99)	0.022
	IQR	0.84 (0.67–1.06)		0.73 (0.56–0.95)	
*MN1*	Cont.	0.99 (0.98–1.00)	0.064	0.99 (0.98–1.00)	0.020
	IQR	0.86 (0.73–1.01)		0.78 (0.63–0.96)	
**Overall Survival, OS**		**HR (95% CI)**	**P**	**HR (95% CI)**	**P**
*FLT3*-ITD AR	Continuous	1.45 (1.03–2.06)	0.035	1.08 (0.98–1.19)	0.133
	≥ 0.5 vs. < 0.5	1.05 (0.56–1.97)	0.879	0.92 (0.47–1.81)	0.807
*NPM1*	Mutated vs. not	1.10 (0.77–1.57)	0.610	1.04 (0.73–1.50)	0.820
*CCNA1*	Cont.	1.00 (1.00–1.00)	< 0.001	1.00 (1.00–1.00)	0.020
	IQR	1.08 (1.03–1.14)		1.07 (1.01–1.13)	
*ERG1*	Cont.	1.04 (1.01–1.07)	0.020	1.04 (1.01–1.07)	0.002
	IQR	1.21 (1.03–1.41)		1.36 (1.12–1.64)	
*EVI1*	Binary	1.59 (1.10–2.29)	0.014	2.03 (1.39–2.96)	< 0.001
	Cont.	1.01 (1.00–1.01)	0.017	1.01 (1.00–1.01)	0.004
	IQR	1.00 (1.00–1.00)		1.00 (1.00–1.00)	
*FLT3*	Cont.	1.02 (1.00–1.03)	0.013	1.02 (1.00–1.03)	0.026
	IQR	1.14 (1.03–1.27)		1.13 (1.01–1.26)	
*IL3RA*	Cont.	1.02 (0.99–1.05)	0.128	1.03 (1.00–1.06)	0.044
	IQR	1.08 (0.98–1.19)		1.11 (1.00–1.22)	
**Relapse-Free Survival, RFS**		**HR (95% CI)**	**P**	**HR (95% CI)**	**P**
*FLT3*-ITD AR	Continuous	0.99 (0.30–3.30)	0.983	1.19 (0.92–1.55)	0.187
	≥ 0.5 vs. < 0.5	0.88 (0.40–1.94)	0.750	1.95 (0.77–4.94)	0.161
*NPM1*	Mutated vs. not	0.96 (0.60–1.52)	0.850	0.96 (0.59–1.54)	0.860
*CCNA1*	Cont.	1.00 (1.00–1.01)	0.004	1.00 (1.00–1.00)	0.083
	IQR	1.20 (1.06–1.36)		1.10 (0.99–1.22)	
*EVI1*	Binary	1.89 (1.14–3.13)	0.014	1.94 (1.13–3.32)	0.015
	Cont.	1.01 (1.00–1.02)	0.004	1.02 (1.01–1.03)	0.003
	IQR	1.00 (1.00–1.00)		1.00 (1.00–1.00)	
*IL3RA*	Cont.	1.02 (1.00–1.05)	0.092	1.03 (1.00–1.07)	0.050
	IQR	1.09 (0.99–1.21)		1.12 (1.00–1.25)	

*FLT3*-ITD AR was analyzed both as a continuous variable (Cont.) and as a binary variable as defined by the ELN-2017 guidelines (≥0.5 vs. < 0.5). Transcript expression fold changes were analyzed both as unadjusted variables (Cont.) and adjusted (divided) by the interquartile range (IQR) of the corresponding expression variable in the discovery data. *EVI1* was analyzed as a continuous and as a binary variable (expressed vs. not)

**Table 2:** Univariate Analyses in the Discovery Cohort

**Variable**		**Complete Response, CR (*N* = 185)**		**Overall Survival, OS (*N*** **= 185)**		**Relapse-Free Survival, RFS (*N*** **= 106)**	
		**OR (95% CI)**	**P**	**HR (95% CI)**	**P**	**HR (95% CI)**	**P**
Age	Continuous	0.96 (0.94–0.98)	< 0.001	1.06 (1.04–1.07)	< 0.001	1.04 (1.02–1.05)	< 0.001
	By Decade	0.66 (0.53–0.82)	< 0.001	1.75 (1.52–2.01)	< 0.001	1.41 (1.20–1.67)	< 0.001
Cytogenetics	Fav. vs. Interm.	5.29 (1.15–24.40)	0.033*	0.24 (0.10–0.59)	0.002*	0.29 (0.12–0.69)	0.005*
	Unfav. vs. Interm.	0.42 (0.18–0.97)	0.043*	1.77 (1.12–2.79)	0.014*	1.38 (0.71–2.68)	0.349*
	Unk. vs. Interm.	0.57 (0.28–1.16)	0.124*	1.25 (0.84–1.88)	0.271*	0.80 (0.46–1.41)	0.446*
Performance Status	Numeric	0.82 (0.58–1.15)	0.246	1.07 (0.89–1.30)	0.462	0.84 (0.62–1.15)	0.278
	2–3 vs. 0–1	0.37 (0.17–0.81)	0.013	1.49 (0.97–2.29)	0.07	1.01 (0.50–2.03)	0.971
Study	S0106 vs S9031	2.88 (1.20–6.87)	0.018*	0.28 (0.17–0.46)	< 0.001*	0.35 (0.18–0.69)	0.002*
	S9333 vs S9031	1.04 (0.40–2.73)	0.934*	0.87 (0.53–1.43)	0.58*	0.79 (0.38–1.67)	0.54*
	S0112 vs S9031	0.63 (0.17–2.33)	0.484*	1.16 (0.61–2.22)	0.645*	0.79 (0.27–2.30)	0.669*
Immunophenotype	2 vs 0	1.37 (0.68–2.75)	0.384	1.14 (0.77–1.70)	0.504	1.26 (0.76–2.08)	0.373
	1 vs 0	0.73 (0.33–1.57)	0.415	1.02 (0.64–1.61)	0.946	0.97 (0.51–1.86)	0.928
AML Onset	Secondary vs. DN	0.23 (0.08–0.67)	0.007	1.89 (1.10–3.25)	0.02	0.95 (0.30–3.01)	0.925
ELN_2017_ in MNCs	Fav. vs. Interm.	3.11 (1.16–8.32)	0.024*	0.58 (0.32–1.02)	0.06*	0.47 (0.24–0.92)	0.027*
	Adv. vs. Interm.	0.64 (0.27–1.56)	0.328*	1.66 (1.00–2.77)	0.05*	1.72 (0.88–3.35)	0.113*
	Unk. vs. Interm.	0.72 (0.29–1.75)	0.467*	1.32 (0.78–2.23)	0.305*	0.76 (0.37–1.54)	0.441*
ELN_2017_ in VLBs	Fav. vs. Interm.	3.69 (1.30–10.52)	0.014*	0.38 (0.21–0.68)	0.001*	0.37 (0.18–0.77)	0.008*
	Adv. vs. Interm.	0.77 (0.30–1.98)	0.587*	1.10 (0.66–1.84)	0.72*	1.45 (0.71–2.97)	0.312*
	Unk. vs. Interm.	0.96 (0.37–2.47)	0.928*	0.78 (0.46–1.33)	0.36*	0.64 (0.31–1.35)	0.242*

* Indicates that the overall *p*-value was significant (< 0.05)

### Performance of novel risk models utilizing ELN and other prognostic factors

Multivariable models for CR, OS, and RFS were developed separately for each cell population using age, ELN-2017 risk group, PS, AML onset, immunophenotype, clinical trial, transcript biomarker and expression as possible covariates (Additional File, Models Details). In the discovery cohort, the models with the best performance were obtained when clinical variables and expression biomarkers were integrated; however, when applied to an independent population of patients in the validation cohort, the performances of integrated models for most outcomes were not superior to AGE + ELN_2017_ models (Table 3). If a model is generalizable to a broad population, AUCs or C-statistics will be nearly equivalent in the two cohorts. Generalizability of the developed integrated models was inconsistent across CR, OS and RFS outcomes.

**Table 3:** Multivariable models for CR, OS and RFS

**Models**	**AUCs or C-statistics**			
	**MNCs**		**VLBs**	
**Complete Response (CR, AUC)**	**Discovery**	**Validation**	**Discovery**	**Validation**
ELN_2017_	0.66	0.73	0.66	0.67
AGE + ELN_2017_	0.71	0.72	0.71	0.72
AGE + PS + *GATA2* + *BAALC* (MNCs)	0.77^a^	0.67	N/A	N/A
AGE + PS + *MN1* + *GATA2* (VLBs)	N/A	N/A	0.72^a^	0.70
**Overall Survival (OS, C Statistic)**	**Discovery**	**Validation**	**Discovery**	**Validation**
ELN_2017_	0.60	0.68	0.59	0.68
AGE + ELN_2017_	0.72	0.70	0.71	0.71
AGE + ELN_2017_ + *ERG1* + *EVI1* + *JAG1* + *JAG1*^a^ELN (MNCs)	0.73^a^	0.69	N/A	N/A
AGE + ELN_2017_ + *EVI1* + *ERG1* + *CCNA1* (VLBs)	N/A	N/A	0.71^a^	0.73
**Relapse-Free Survival (RFS, C Statistic)**	**Discovery**	**Validation**	**Discovery**	**Validation**
ELN_2017_^b^	0.60	0.54	0.61	0.54
AGE + ELN_2017_^b^	0.67	0.63	0.66	0.62
AGE + ELN_2017_^b^ + *EVI1* + *CCNA1* (MNCs)	0.69^a^	0.65	N/A	N/A
AGE + ELN_2017_^b^ + *EVI1* + *CCNA1* (VLBs)	N/A	N/A	0.72^a^	0.65

^a^AUCs or C-statistics for the integrated models come from cross-validation. 1/5 of the discovery data were used to build a model, and the remaining 4/5 of the data were fit to this model, resulting in five C-statistics or AUCs. The mean of the five is presented in the table

^b^Due to small sample size, ELN_2017_ in the RFS models was categorized into adverse vs. not adverse (including intermediate, favorable, and unknown)

**Fig. 1:**
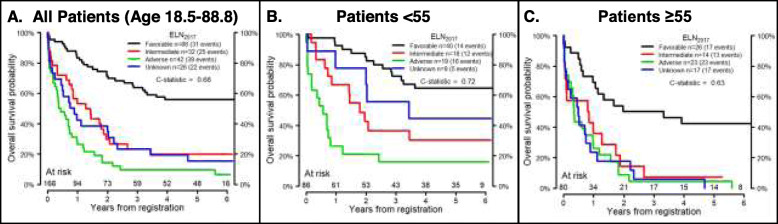
Performance of ELN_2017_ model in Mononuclear cells. Overall Survival probability over time by ELN_2017_ risk group in MNCs from the validation cohort (*n* = 166). C-statistics are for the ELN_2017_ model fit to the validation cohort for all patients (age 18.5–88.8, **a**), patients younger than 55 years old (*N* = 86, **b**), and patients 55 years and older (*N* = 80, **c**). The total number of patients who were at risk of death (alive and uncensored) are shown for each year of follow-up

**Fig. 2:**
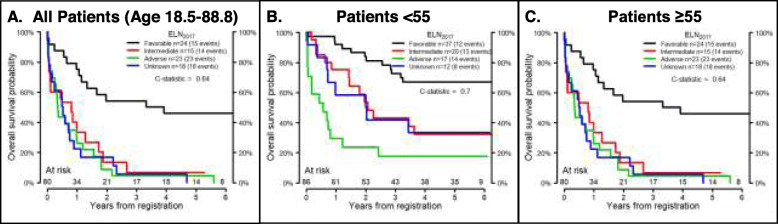
Performance of ELN_2017_ model in Viable Leukemic Blasts. Overall Survival probability over time by ELN_2017_ risk group in VLBs from the validation cohort (*n* = 166). C-statistics are for the ELN_2017_ model fit to the validation cohort for all patients (age 18.5–88.8, **a**), patients younger than 55 years old (*N* = 86, **b**), and patients 55 years and older (*N* = 80, **c**). The total number of patients who were at risk of death (alive and uncensored) are shown for each year of follow-up

**Fig. 3:**
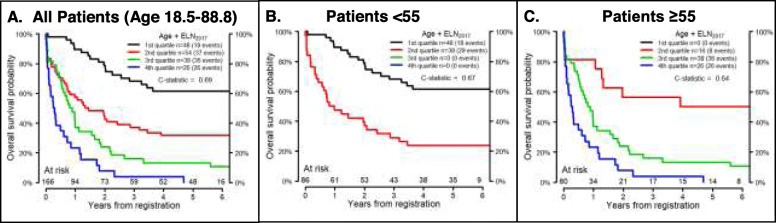
Performance of AGE + ELN_2017_ Model in Mononuclear Cells. Overall Survival probability over time as predicted by the AGE + ELN_2017_ models developed using the discovery cohort in MNCs. The continuous risk score from the AGE + ELN_2017_ model in the discovery cohort was divided into quartiles and the boundaries of these quartiles were used to define a four-level categorical variable. A model was fit using this categorical variable in the validation cohort for all patients (*N* = 166, age 18.5–88.8, **a**), patients younger than 55 years old (*N* = 86, **b**), and patients 55 years and older (*N* = 80, **c**). There were no patients younger than 55 in 3rd and 4th quartiles (**b**) or patients older than 55 in 1st quartile (**c**). The total number of patients who were at risk of death (alive and uncensored) are shown for each year of follow-up

**Fig. 4:**
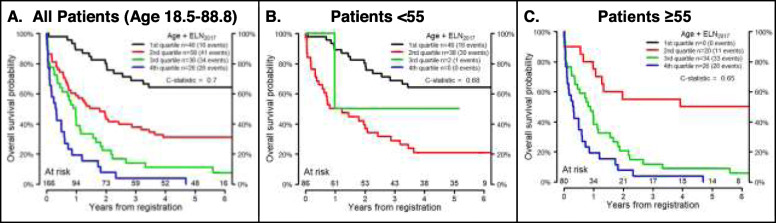
Performance of AGE + ELN_2017_ Model in Viable Leukemic Blasts. Overall Survival probability over time as predicted by the AGE + ELN_2017_ models developed using the discovery cohort in MNCs. The continuous risk score from the AGE + ELN_2017_ model in the discovery cohort was divided into quartiles and the boundaries of these quartiles were used to defined a four-level categorical variable. A model was fit using this categorical variable in the validation cohort for all patients (*N* = 166, age 18.5–88.8, **a**), patients younger than 55 years old (*N* = 86, **b**), and patients 55 years and older (*N* = 80, **c**). There were no patients younger than 55 in 4th quartiles (**b**) or patients older than 55 in 1st quartile (**c**). The total number of patients who were at risk of death (alive and uncensored) are shown for each year of follow-up

The ELN_2017_ model divides patients into 4 groups: favorable, intermediate, adverse, and unknown. Figure 1 shows OS by ELN_2017_ risk in MNCs and VLBs from the validation cohort. Since previous studies demonstrated a worse prognosis for intermediate risk patients over the age of 55 [8, 44], the ELN_2017_ models were also applied to younger (age < 55) and older (age ≥ 55) patients. ELN_2017_ models were a better fit for the younger patients, whether using data derived from MNCs (Fig. 1) or VLBs (Fig. 2). To visualize the AGE + ELN_2017_ model for OS, the continuous risk score from the AGE + ELN_2017_ model in the discovery data was divided into quartiles to parallel the ELN_2017_ model, and boundaries of these quartiles were applied to the validation data (Figs. 3 and 4). Though these plots are intended to be exploratory, the quartiles defined by the AGE + ELN_2017_ models visually show more separation between curves than do the ELN_2017_ risk groups in MNCs and in VLBs (Figs. 3a and 4a vs Figs. 1a and 2a). The c-statistics for the AGE + ELN_2017_ models are also slightly higher than the c-statistics for the ELN_2017_ models. There were no patients younger than 55 in the quartiles representing the poorest outcomes (3rd and 4th quartiles in MNCs and 4th quartile in VLBs) and no patients older than 55 in the 1st quartile, representing the best outcomes. This is due to the fact that older age was associated with poorer outcomes in the multivariable models controlling for ELN risk and age, and these models were used to derive the quartiles in the figure.

### Evaluation of simplified ELN-2017 and AGE + ELN_2017_ models

To investigate the impact of *ASXL1*, *CEBPA*, *RUNX1* and *TP53* mutations on risk stratification, we evaluated the performance of modified models that did not include mutation data for these 4 genes without age (ELN_2017-MOD_) and with age (AGE + ELN_2017-MOD_). Exclusion of mutation status of these four genes resulted in an overall reassignment of risk groups for 46 patients in MNCs and 44 patients in VLBs of the 351 patients (Additional File, Table S8). Both models were developed using the discovery data from the MNCs and VLBs. In the validation cohort, the AUCs and C-statistics were similar between the ELN_2017_ and ELN_2017-MOD_ models, allowing comparable population risk prediction at the community sites that may not have access to genomic mutation screening. Furthermore, the AGE + ELN_2017-MOD_ models had almost the exact same performance characteristics as the AGE + ELN_2017_ models (Table 4).

**Table 4:** Performance of simplified ELN-2017 risk stratification criteria

**Covariates**	**Discovery**	**Validation**
**Complete Response (CR, AUC)**		
ELN_2017_	0.65	0.74
AGE + ELN_2017_	0.71	0.76
ELN_2017-MOD_	0.65	0.72
AGE + ELN_2017-MOD_	0.71	0.77
**Overall Survival (OS, C Statistic)**		
ELN_2017_	0.60	0.68
AGE + ELN_2017_	0.71	0.73
ELN_2017-MOD_	0.60	0.67
AGE + ELN_2017-MOD_	0.72	0.73
**Relapse-Free Survival (RFS, C Statistic)**		
ELN_2017_	0.64	0.62
AGE + ELN_2017_	0.68	0.67
ELN_2017-MOD_	0.62	0.63
AGE + ELN_2017-MOD_	0.67	0.68

Comparison of predictive value of ELN_2017_ and ELN_2017-MOD_ and change in predictive value with the addition of age as a covariate

## Discussion

Risk stratification of AML patients enables physicians to triage patients for optimal therapy. Many prognostic factors have been identified, but relatively few have made their way into clinical practice. The revised ELN-2017 guidelines combine cytogenetic abnormalities and genetic mutations to stratify patients with AML into favorable, intermediate, and adverse risk groups [1]. We examined the predictive accuracy of risk models developed using the ELN-2017 guidelines with and without incorporating additional prognostic factors, as well as how these models performed in VLBs and MNCs. ELN_2017_ predictive models were a better fit for younger patients. Models utilizing the expression results from selected transcript biomarkers did not provide substantial improvement over the ELN_2017_ models, regardless of whether transcripts were tested in MNCs or VLBs. In addition, we examined the potential contribution of mutations in *ASXL1*, *CEBPA*, *RUNX1* and *TP53,* given that clinical testing for these genes may not be readily available in many settings. The ELN_2017-MOD_ demonstrated that while these mutations may provide additional benefit for risk stratification of individual patients, their contribution to the predictive accuracy of the ELN_2017_ models  was limited in our cohorts of AML patients. Thus, ELN-2017 guidelines remain a critical tool for risk stratifying AML patients, but the findings illustrate the need for additional prognostic factors to improve risk stratification, especially in older adults with AML. Moreover, observations suggest that risk stratification models may need to be developed separately for older patients.

Previous studies have examined the performance of ELN-2017 guidelines in patients with AML [2–4, 10]. Similar to our observation in patients younger than 55, authors confirmed the prognostic significance of the ELN-2017 guidelines, with patients stratified as favorable, intermediate, and adverse having a 5-year OS of approximately 60, 40, and 20%, respectively [2–4]. Our results are consistent with the previous report that ELN-2017 guidelines are not as informative for older adults, supporting the need for additional studies for this population [10]. Age remains one of the most robust prognostic factors for patients with AML. As demonstrated in Table 3, AGE models performed comparably to ELN_2017_ models, with the AGE + ELN_2017_ models displaying the best performances. Given that a majority of patients diagnosed with AML are over the age of 65 [45], current risk stratification guidelines for patients with AML need to be adjusted for age or recalibrated for older patients. Similarly, the findings demonstrate the need for more accurate risk stratification models for older adults with AML. Such models will likely require the inclusion of novel prognostic biomarkers.

Although age-related comorbidities and differences in therapy play a role in the poor outcomes for older adults with AML, these factors cannot fully explain the higher relapse rates for these patients [7, 8]. Older adults with AML frequently harbor mutations in genes associated with the spliceosome, methylation and chromatin remodeling, which are commonly identified in patients with MDS or secondary AML [10, 12, 46–49]. This age-related mutational profile, as well as unknown molecular factors, may contribute to the resistant biology that leads to higher relapse rates and an adverse prognosis for older adults with AML. The integration of age into prognostic models partially compensated for some of the age-related adverse biology. This approach, however, cannot fully account for the intra- and inter-patient heterogeneity in AML blasts from older adults, and as such, remains a relatively imprecise surrogate for the biological factors causing resistance in older patients. Investigations into the biology governing resistance in older adults with AML are warranted to elucidate the molecular factors responsible for the poor outcomes.

The ELN recently integrated mutations in *ASXL1*, *RUNX1,* and *TP53* into their guidelines. In addition, the ELN-2017 guidelines now require double *CEBPA* mutations for patients to be deemed favorable risk. These changes require either a part of or the entire reading frame of genes to be sequenced. Such sequencing technology is either not available or may be cost-prohibitive in many areas. To better understand the prognostic benefit of these changes, we evaluated the performance of a modified model (ELN_2017-MOD_), which excluded the mutation data for *ASXL1*, *RUNX1*, *TP53* and *CEBPA*. The ELN_2017-MOD_ had a similar performance to the ELN_2017_ model. Inclusion of age into the model (AGE + ELN_2017-MOD_) demonstrated an improved performance over the ELN_2017-MOD_ model. While a small number of patients changed risk group between the ELN_2017_ and ELN_2017-MOD_ models, the incremental improvement does not negate the potential individual prognostic value of these additional mutations.

The studies also examined the prognostic impact of testing biomarkers in a more homogenous cell population (i.e., VLBs). The concordance in dichotomous mutation calls was almost 100% between MNCs and VLBs. Assays employed to detect mutation (fragment analyses PCR for *FLT3*-ITD and targeted deep sequencing for *ASXL1*, *CEBPA*, *NPM1*, *RUNX1* and *TP53*) paralleled those currently used in clinical testing. However, the sequencing depth of the experiments were not intended to detect very low mutation loads, and as such, sequencing at higher depths may have yielded different results. Unlike dichotomous results, the *FLT3*-ITD AR was higher in VLBs than MNCs, resulting in a shift of the risk classification for 19 patients. Nevertheless, these differences in risk classification did not markedly impact the prognostic significance of the biomarker by itself or when incorporated into models. The transcript biomarkers were primarily selected based on their reported promise as prognostic biomarkers, and some previously validated transcript biomarkers, such as those involving leukemia stem cell signatures, were not examined [50–53]. Similarly, we assayed expression of select transcripts via q-RT/PCR due to the focused nature of the studies and global transcription profiling was not performed. Although expression of the examined transcripts in VLBs did not markedly improve the predictive accuracy of the models, the analyses confirmed that expression of the transcript biomarkers significantly differs between MNCs and VLBs, with most transcripts being expressed at higher levels in the VLBs. Therefore, it remains unclear whether examining VLBs may or may not provide a mechanism to identify novel prognostic biomarkers or improve the prognostic performance of other transcript biomarkers. Studies are currently underway to examine these questions using a more comprehensive approach, which includes global RNA sequencing of the MNCs and VLBs.

Although the current report represents the largest analysis of paired MNCs and VLBs from AML patients, the number and source (i.e., BM vs. PB) of samples may limit the ability to detect significant differences between models utilizing MNCs vs. VLBs. The number of examined patients was limited by the availability of specimens with adequate vials and the resources. Nevertheless, the data suggest that prognostic biomarkers (e.g., *FLT3*-ITD AR) yield different results depending upon the examined material (i.e., MNCs vs. VLBs) and highlight the need to identify additional biomarkers to improve current risk stratification guidelines. Unfortunately, large numbers of paired diagnostic BM and PB samples are not readily available for correlative studies to evaluate the impact of specimen source. However, some comparisons between MNCs from paired BM and PB have been performed by our group and others. While some report potential functional differences [54], others found subtle differences between the two tissue sources [55, 56]. Our previous examination of transcript and mutation biomarkers in paired BM and PB samples did not find any significant differences between unsorted MNCs from the PB versus BM with respect to the immunophenotype of leukemic blasts, mutation detection in *FLT3* and *NPM1* genes, relative quantities of mutations (allelic ratio of *FLT3*-ITD and *NPM1* insertions), or the expression of majority of specific transcripts reported in this paper [6]. These additional biomarker studies will likely require investigations into previously untapped molecular components driving the biology of AML such as the proteome. As a means to improve the homogeneity of treatment, the study examined only those patients who received intensive chemotherapy with curative intent as part of SWOG trials. Despite randomization, the more recent trials were better represented in evaluated populations than older trials, however, the treatment regimens were comparable among the four trials from which the patients were drawn. Thus, the results may not be generalizable to patients receiving therapy outside of evaluated clinical trials, low-intensity regimens (e.g., azacytidine), or targeted agents (e.g., midostaurin). Nonetheless, some recent biomarker studies suggest that previously recognized prognostic factors remain highly informative and predictive for responses to more “targeted’ agents [57–59], and as such, there likely remains some role for the identification of prognostic biomarkers that are applicable across a variety of therapies.

## Conclusions

In summary, this study represents the largest systematic evaluation of prognostic biomarkers in paired MNC and VLB from patients with AML. Overall, the ELN-2017 guidelines risk stratified younger adults with AML more accurately than older adults with AML. In addition, models developed utilizing ELN-2017 guidelines and other selected biomarkers did not substantially improve risk stratification. Similarly, the performance of these models was not significantly impacted by the source of material examined, (i.e., MNC vs. VLB).

## Supplementary information

**Additional file 1: Table S1.** Targeted Sequencing Details. **Table S1A.** Target regions for Wafergen Sequencing. **Table S1B** Primers and Amplicon Details for Wafergen Sequencing. **Table S1C.** Primers for CEBPA targeted MiSeq Assay. **Table S2.** Loci that Failed Quality Control. **Table S3.** Characteristics of selected and unselected SWOG patients. **Fig. S1.** Comparison of Performance Characteristics of selected and unselected SWOG patients. **Table S4.** Characteristics of SWOG patients in the discovery and validation cohorts. **Fig. S2.** Comparison of Performance Characteristics of SWOG patients in the discovery and validation cohorts. **Table S5.** Mutation distribution in discovery and validation cohorts. **Fig. S3.** Mutation Frequency (OncoPrint). **Table S6.** Expression fold change differences between MNCs and VLBs in discovery cohort. **Table S7.** Univariate analyses results, non-significant findings Models Details. **Table S8.** ELN_2017_ versus ELN_2017_-MOD risk assignment in MNCs and VLBs.

## Data Availability

The datasets used and/or analyses used in the current study are available from the corresponding author on reasonable request. Clinical data pertaining to individual patients are available from SWOG upon request and execution of a data use agreement per SWOG and NCI policy.

## Abbreviations

Adv.: Adverse
AIC: Aikike Information Criterion
AML: Acute myeloid leukemia
ANC: Absolute neutrophil count
AR: Allelic ratio
AUC: Area under the Receiver Operating Characteristic curve
BCCA: British Columbia Cancer Agency
BWA: Burrows-Wheeler Aligner
Cont.: Continuous
CR: Complete remission
DAPI: 4′,6-diamidino-2-phenylindole
DN: De novo
DNA: Deoxyribonucleic acid
ELN: European LeukemiaNet
FAB: French-American-British
FACS: Fluorescence-activated cell sorting
Fav.: Favorable
FC: Fold Change
GATK: Genome Analysis Toolkit
Interm.: Intermediate
IP: Immunophenotype
IQR: Interquartile range
ITD: Internal tandem duplication
MNCs: Mononuclear Cells
MOD: Modified
OS: Overall survival
PS: Performance status
QC: Quality control
RFS: Relapse-free survival
RNA: Ribonucleic acid
Ukn.: Unknown
Unfav.: Unfavorable
VAF: Variant allele frequency
VLBs: Viable leukemic blasts
