# Supplementary material for: AML risk stratification models utilizing ELN-2017 guidelines and additional prognostic factors: a SWOG report
Source: Biomark Res. 2020 Aug 12;8:29. doi: 10.1186/s40364-020-00208-1 (PMC7425159; doi:10.1186/s40364-020-00208-1)
Supplement: Supplementary file 1 — Additional file 1: Table S1. Targeted Sequencing Details. Table S1A. Target regions for Wafergen Sequencing. Table S1B Primers and Amplicon Details for Wafergen Sequencing. Table S1C. Primers for CEBPA targeted MiSeq Assay. Table S2. Loci that Failed Quality Control. Table S3. Characteristics of selected and unselected SWOG patients. Fig. S1. Comparison of Performance Characteristics of selected and unselected SWOG patients. Table S4. Characteristics of SWOG patients in the discovery and validation cohorts. Fig. S2. Comparison of Performance Characteristics of SWOG patients in the discovery and validation cohorts. Table S5. Mutation distribution in discovery and validation cohorts. Fig. S3. Mutation Frequency (OncoPrint). Table S6. Expression fold change differences between MNCs and VLBs in discovery cohort. Table S7. Univariate analyses results, non-significant findings Models Details. Table S8. ELN2017 versus ELN2017-MOD risk assignment in MNCs and VLBs. [file 40364_2020_208_MOESM1_ESM.docx]

**AML Risk Stratification Models Utilizing ELN-2017 Guidelines and Additional Prognostic Factors: A SWOG Report**

Era L. Pogosova-Agadjanyan, Anna Moseley, Megan Othus, Frederick R. Appelbaum, Thomas R. Chauncey, I-Ming L. Chen, Harry P. Erba, John E. Godwin, Isaac C. Jenkins, Min Fang, Mike Huynh, Kenneth J. Kopecky, Alan F. List, Jasmine Naru, Jerald P. Radich, Emily Stevens, Brooke E. Willborg, Cheryl L. Willman, Brent L. Wood, Qing Zhang, Soheil Meshinchi, and Derek L. Stirewalt

**Additional File**

Supplemental Methods: Wafergen Sequencing page 2

Table S1: Targeted Sequencing Details pages 3-5

Table S1A: Target regions for Wafergen Sequencing page 3

Table S1B: Primers and Amplicon Details for Wafergen Sequencing page 4

Table S1C: Primers for CEBPA targeted MiSeq Assay page 5

Table S2. Loci that Failed Quality Control page 6

Table S3: Characteristics of selected and unselected SWOG patients page 7

Fig. S1: Comparison of Performance Characteristics of selected page 9

and unselected SWOG patients

Table S4: Characteristics of SWOG patients in the discovery and validation cohorts page 10

Fig. S2: Comparison of Performance Characteristics of SWOG patients in the page 12

discovery and validation cohorts.

Table S5: Mutation distribution in discovery and validation cohorts page 13

Fig. S3: Mutation Frequency (OncoPrint) page 14

Table S6: Expression fold change differences between MNCs and VLBs page 15

in discovery cohort

Table S7: Univariate analyses results, non-significant findings page 16

Models Details page 18

Table S8: ELN_2017_ versus ELN_2017_-MOD risk assignment in MNCs and VLBs page 23

**Supplemental Methods: Wafergen Sequencing**

A target list of gene targets was provided to Wafergen as input for primer design. Redundant primer assays were added for difficult target regions where the selected assays were not optimal such as where the amplicon lengths were long or where multiple hits were produced. Primers were bioinformatically and manually verified to ensure all target regions were covered. Wafergen compatible adapters were added to the primer sequences and the oligos targeted PCR products that were 203-500bp in length.

Primers were synthesized by IDT (Integrated DNA Technologies, USA) and normalized to a working stock of 1.25uM. PCR reactions were set up against samples using the Seq-Ready™ TE MultiSample System (Wafergen, USA) with Seq-Ready™ TE MultiSample FLEX Kits (Wafergen, USA) according to manufacturer’s specifications. Brew containing template and brew containing primers were dispensed into 384-well plates using the Janus liquid handling system (Perkin Elmer, USA). DNA samples and PCR reagents from the 384-well brew plates were dispensed into nanowell SmartChips using the MultiSample NanoDispenser (Wafergen, USA). The SmartChips were amplified on a SmartChip™ TE Cycler (Wafergen, USA) using the manufacturer’s COT40 program with 40 cycles of amplification. The amplified products were pooled and purified with Ampure XP SPRI beads.

Samples were sequenced on the Illumina MiSeq platform at PET250 read length, using V2 chemistry, as per manufacturer’s instructions.

**Table S1: Targeted Sequencing Details**

**Table S1A: Target regions for Wafergen Sequencing**

| **Target** | **RefSeqID** | **Gene** | **Chr.** | **Start** | **Stop** |
| --- | --- | --- | --- | --- | --- |
| ASXL1 | NM_015338 | ASXL1 | 20 | 31022234 | 31027122 |
| ASXL1 | NM_001164603 | ASXL1-SV | 20 | 30959966 | 30960352 |
| CEPBA | NM_004364.3 | CEBPA | 19 | 33790839 | 33793430 |
| RUNX1 | NM_001754 | RUNX1 | 21 | 36231770 | 36231875 |
| RUNX1 | NM_001754 | RUNX1 | 21 | 36252853 | 36253010 |
| RUNX1 | NM_001754 | RUNX1 | 21 | 36259139 | 36259393 |
| RUNX1 | NM_001754 | RUNX1 | 21 | 36265221 | 36265260 |
| RUNX1 | NM_001754 | RUNX1 | 21 | 36160097 | 36164907 |
| TP53 | NM_00546.5 | TP53 | 17 | 7571719 | 7573008 |
| TP53 | NM_00546.5 | TP53 | 17 | 7573926 | 7574033 |
| TP53 | NM_00546.5 | TP53 | 17 | 7576852 | 7576926 |
| TP53 | NM_00546.5 | TP53 | 17 | 7577018 | 7577155 |
| TP53 | NM_00546.5 | TP53 | 17 | 7577498 | 7577608 |
| TP53 | NM_00546.5 | TP53 | 17 | 7578176 | 7578289 |
| TP53 | NM_00546.5 | TP53 | 17 | 7578370 | 7578554 |
| TP53 | NM_00546.5 | TP53 | 17 | 7579311 | 7579590 |
| TP53 | NM_00546.5 | TP53 | 17 | 7579699 | 7579721 |
| TP53 | NM_00546.5 | TP53 | 17 | 7579838 | 7579940 |
| TP53 | NM_00546.5 | TP53 | 17 | 7590694 | 7590868 |
| TP53 | NM_001276698 | TP53-SV | 17 | 7571719 | 7573008 |
| TP53 | NM_001276698 | TP53-SV | 17 | 7573926 | 7574033 |
| TP53 | NM_001276698 | TP53-SV | 17 | 7576524 | 7576657 |
| TP53 | NM_001276698 | TP53-SV | 17 | 7576852 | 7576926 |
| TP53 | NM_001276698 | TP53-SV | 17 | 7577018 | 7577155 |
| TP53 | NM_001276698 | TP53-SV | 17 | 7577498 | 7577608 |
| TP53 | NM_001276698 | TP53-SV | 17 | 7578176 | 7578289 |
| TP53 | NM_001276698 | TP53-SV | 17 | 7578370 | 7578811 |

**Table S1B: Primers and Amplicon Details for Wafergen Sequencing**


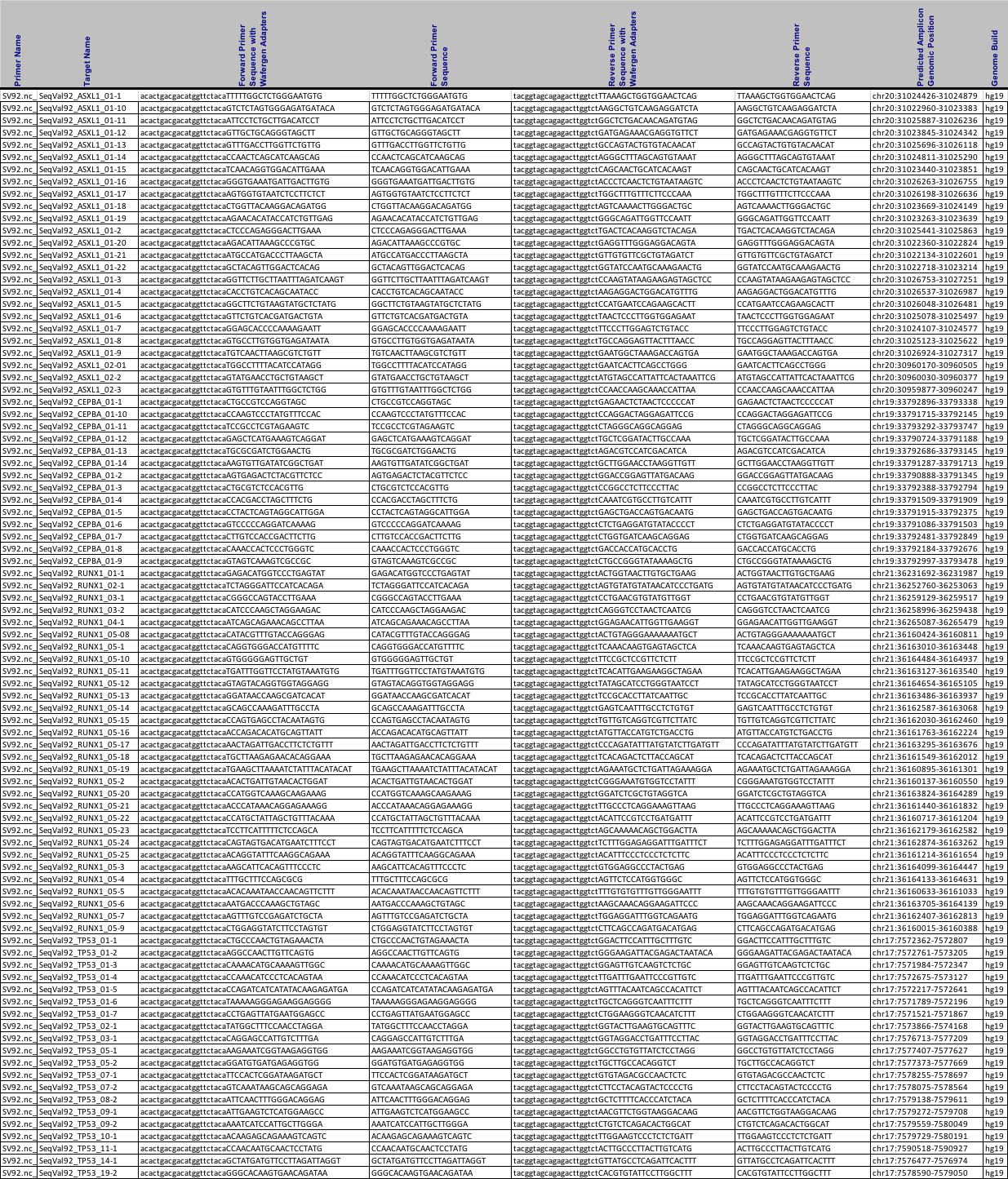


**Table S1C: Primers for CEBPA targeted MiSeq Assay**

| **Primer Name** | **Primer Sequence (5'-3')** |
| --- | --- |
| CEBPA-p1-F | AGCAGGGTCTCCGGGTGGGCGG |
| CEBPA-p1-R | CGGCTGTGCTGGAACAGGTCGGC |
| CEBPA-p2-F | GCACGAGACGTCCATCGACATCAGC |
| CEBPA-p2-R | AGCTGCTTGGCTTCATCCTCCTCGC |
| CEBPA-p3-F | TGGACGGCAGGCTGGAGCCCCTGTA |
| CEBPA-p3-R | CAGCCCCTTGAGCGCGCTGCCA |
| CEBPA-p4-F | ACGCCCGTGCCCAGCCCGCAC |
| CEBPA-p4-R | GCAGCTGGCGGAAGATGCCCCGCA |
| CEBPA-p5-F | ACAATGACCGCCTGCGCAAGCGGGT |
| CEBPA-p5-R | CCCAGCTCAGCCCCAAGAATTCTCC |

**Table S2. Loci that Failed Quality Control**

|  |  |  |  |  |  |  | Cosmic Mutation Frequency | | |
| --- | --- | --- | --- | --- | --- | --- | --- | --- | --- |
| Gene | Genomic Start | Genomic End | Start | End | Exon | Accession | MS | FS | SG |
| ASXL1 | chr20:31,022,343 | chr20:31,022,413 | G610 | H633 | Exon 12 | NM_015338 | 24 | 10 | 4 |
| RUNX1 | chr21:36,259,363 | chr21:36,259,409 | D33 | P43 | Exon 4 | NM_001754 | 0 | 1 | 0 |
| RUNX1 | chr21:36,164,579 | chr21:36,164,720 | P386 | C432 | Exon 9 | NM_001754 | 3 | 0 | 4 |

Genomic mutation frequency for missense (MS), frame shift (FS) and stop/gain (SG) mutations in myeloid malignancies based on Cosmic Database

**Table S3: Characteristics of selected and remaining SWOG patients**

| **Characteristic** | | **Total**  **(N=1042)** | | **Included**  **(N=351)** | | **Not Included (N=691)** | | **P*** |
| --- | --- | --- | --- | --- | --- | --- | --- | --- |
|  |  | **No.** | | **No.** | | **No.** | |  |
| Age (yrs) | median | 57.1 | | 56.3 | | 57.4 | | 0.211 |
|  | Range | 18.5 -88.8 | | 18.5 -88.8 | | 18.7 -85.1 | |  |
|  | N Missing | 0 | | 0 | | 0 | |  |
| Marrow blasts (%) | median | 65 | | 71 | | 60 | | <.0001 |
|  | Range | 0 -100 | | 7 -100 | | 0 -100 | |  |
|  | N Missing | 41 | | 11 | | 30 | |  |
| WBC (10^9/L) | median | 11.6 | | 36.5 | | 6.2 | | <.0001 |
|  | Range | 0.2 -545 | | 0.7 -369.7 | | 0.2 -545 | |  |
|  | N Missing | 0 | | 0 | | 0 | |  |
| PB Blasts (%) | median | 27 | | 41 | | 21 | | <.0001 |
|  | Range | 0 -99 | | 0 -99 | | 0 -99 | |  |
|  | N Missing | 62 | | 10 | | 52 | |  |
| PB Blasts (10^9/L) | median | 3.2 | | 10.1 | | 1.2 | | <.0001 |
|  | Range | 0 -490.5 | | 0 -347.5 | | 0 -490.5 | |  |
|  | N Missing | 62 | | 10 | | 52 | |  |
| Neutrophils (%) | median | 11 | | 8 | | 13 | | <.0001 |
|  | Range | 0 -97 | | 0 -96 | | 0 -97 | |  |
|  | N Missing | 31 | | 4 | | 27 | |  |
| ANC (10^9/L) | median | 1 | | 2.1 | | 0.7 | | <.0001 |
|  | Range | 0 -171.6 | | 0 -106.9 | | 0 -171.6 | |  |
|  | N Missing | 31 | | 4 | | 27 | |  |
| Hg (g/dL) | median | 9.1 | | 9.1 | | 9.1 | | 0.275 |
|  | Range | 3.5 -29.1 | | 4.4 -29.1 | | 3.5 -28.5 | |  |
|  | N Missing | 18 | | 8 | | 10 | |  |
| PLT (10^9/L) | median | 54 | | 52.5 | | 55 | | 0.336 |
|  | Range | 2 -9300 | | 6 -449 | | 2 -9300 | |  |
|  | N Missing | 3 | | 1 | | 2 | |  |
| Registration year | median | 2006 | | 2005 | | 2006 | | 0.6060 |
|  | Range | 1992 -2009 | | 1992 -2009 | | 1992 -2009 | |  |
|  | N Missing | 0 | | 0 | | 0 | |  |
| **Characteristic** | | **No.** | **%** | **No.** | **%** | **No.** | **%** | **P*** |
| Sex | Female | 483 | 46% | 169 | 48% | 314 | 45% | 0.408 |
|  | Male | 559 | 54% | 182 | 52% | 377 | 55% |  |
| Race | White | 897 | 86% | 291 | 83% | 606 | 88% | 0.042 |
|  | Black | 74 | 7% | 37 | 11% | 37 | 5% |  |
|  | Asian | 33 | 3% | 11 | 3% | 22 | 3% |  |
|  | Hawaiian or Pacific Islander | 4 | 0% | 0 | 0% | 4 | 1% |  |
|  | Am. Indian or Alaska Native | 7 | 1% | 2 | 1% | 5 | 1% |  |
|  | Unknown | 27 | 3% | 10 | 3% | 17 | 2% |  |
| Hispanic ethnicity | Yes | 39 | 4% | 14 | 4% | 25 | 4% | 0.035 |
|  | No | 893 | 86% | 288 | 82% | 605 | 88% |  |
|  | Unknown | 110 | 11% | 49 | 14% | 61 | 9% |  |
| Secondary AML | No | 934 | 90% | 322 | 92% | 612 | 89% | 0.113 |
|  | Yes | 108 | 10% | 29 | 8% | 79 | 11% |  |
| Cytogenetics | -5/5q-,-7/7q- | 102 | 10% | 22 | 6% | 80 | 12% | 0.003 |
|  | CBF | 86 | 8% | 40 | 11% | 46 | 7% |  |
|  | Normal | 326 | 31% | 127 | 36% | 199 | 29% |  |
|  | Normal+Nonclonal | 18 | 2% | 7 | 2% | 11 | 2% |  |
|  | Other | 234 | 22% | 76 | 22% | 158 | 23% |  |
|  | Missing | 276 | 26% | 79 | 23% | 197 | 29% |  |
| FAB class | M0 | 67 | 6% | 22 | 6% | 45 | 7% | <.0001 |
|  | M1 | 234 | 22% | 75 | 21% | 159 | 23% |  |
|  | M2 | 292 | 28% | 83 | 24% | 209 | 30% |  |
|  | M3 | 3 | 0% | 1 | 0% | 2 | 0% |  |
|  | M4/M4eos | 251 | 24% | 111 | 32% | 140 | 20% |  |
|  | M5 | 125 | 12% | 47 | 13% | 78 | 11% |  |
|  | M6 | 27 | 3% | 1 | 0% | 26 | 4% |  |
|  | M7 | 16 | 2% | 1 | 0% | 15 | 2% |  |
|  | Other | 12 | 1% | 4 | 1% | 8 | 1% |  |
|  | Missing | 15 | 1% | 6 | 2% | 9 | 1% |  |
| Performance status | 0 | 344 | 33% | 108 | 31% | 236 | 34% | 0.235 |
|  | 1 | 495 | 48% | 166 | 47% | 329 | 48% |  |
|  | 2 | 125 | 12% | 48 | 14% | 77 | 11% |  |
|  | 3 | 66 | 6% | 28 | 8% | 38 | 5% |  |
|  | Missing | 12 | 1% | 1 | 0% | 11 | 2% |  |
| Clinical trial | S0106 | 597 | 57% | 206 | 59% | 391 | 57% | 0.013 |
|  | S0112 | 60 | 6% | 23 | 7% | 37 | 5% |  |
|  | S9031 | 224 | 21% | 57 | 16% | 167 | 24% |  |
|  | S9333 | 161 | 15% | 65 | 19% | 96 | 14% |  |
| Complete response | No | 433 | 42% | 141 | 40% | 292 | 42% | 0.518 |
|  | Yes | 609 | 58% | 210 | 60% | 399 | 58% |  |

Abbreviations: WBC, white blood cell count; PB, peripheral blood; ANC, absolute neutrophil count; Hg, hemoglobin; PLT, platelet count; AML, acute myeloid leukaemia; FAB, French American British classification.

*P-value from Wilcoxon rank-sum tests for continuous variables and chi-squared or Fisher’s exact tests for categorical variables, depending on the frequency distribution.

**Fig. S1: Comparison of Survival Outcomes of selected and unselected SWOG patients.**

**B. Overall Survival**


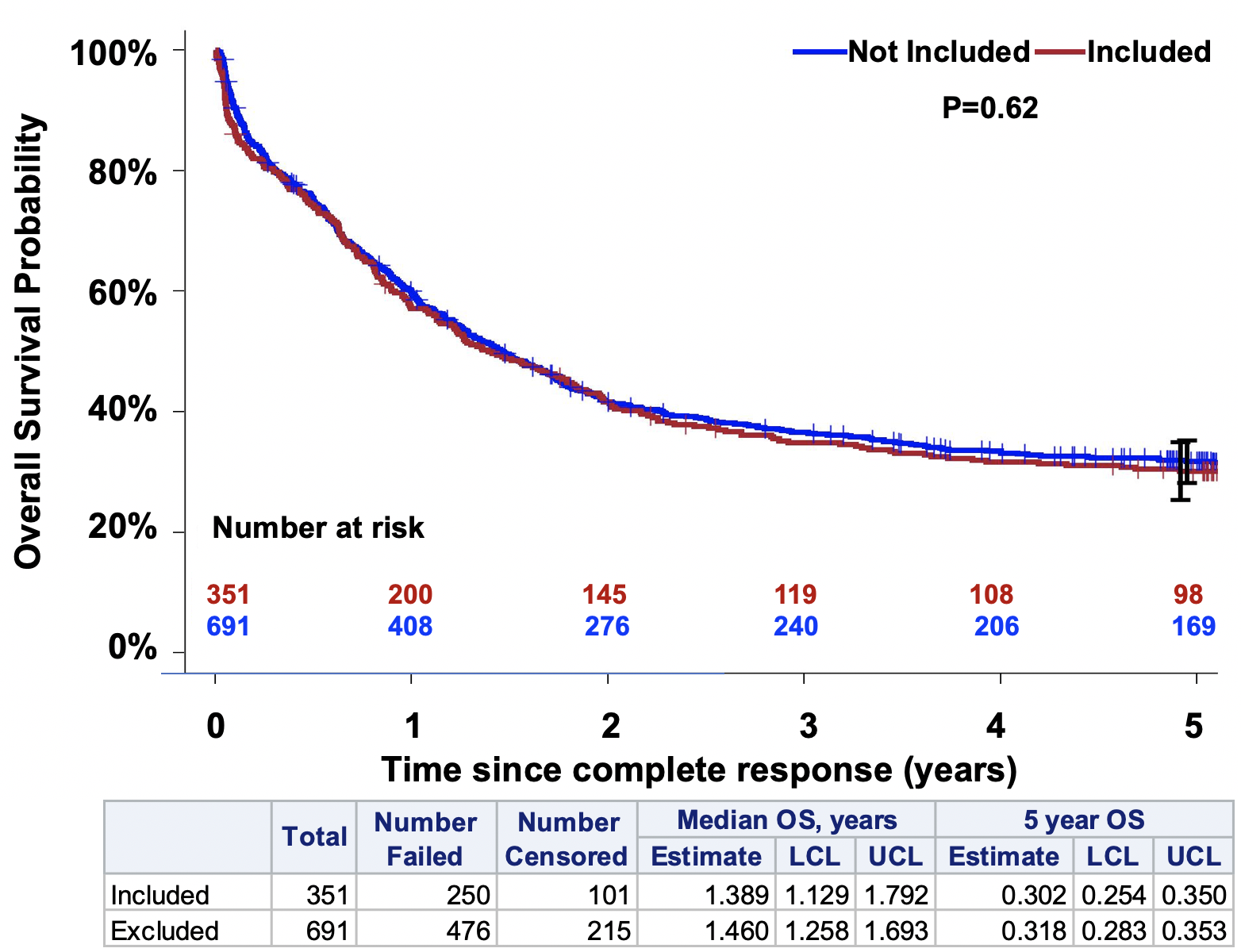


**A. Relapse Free Survival**


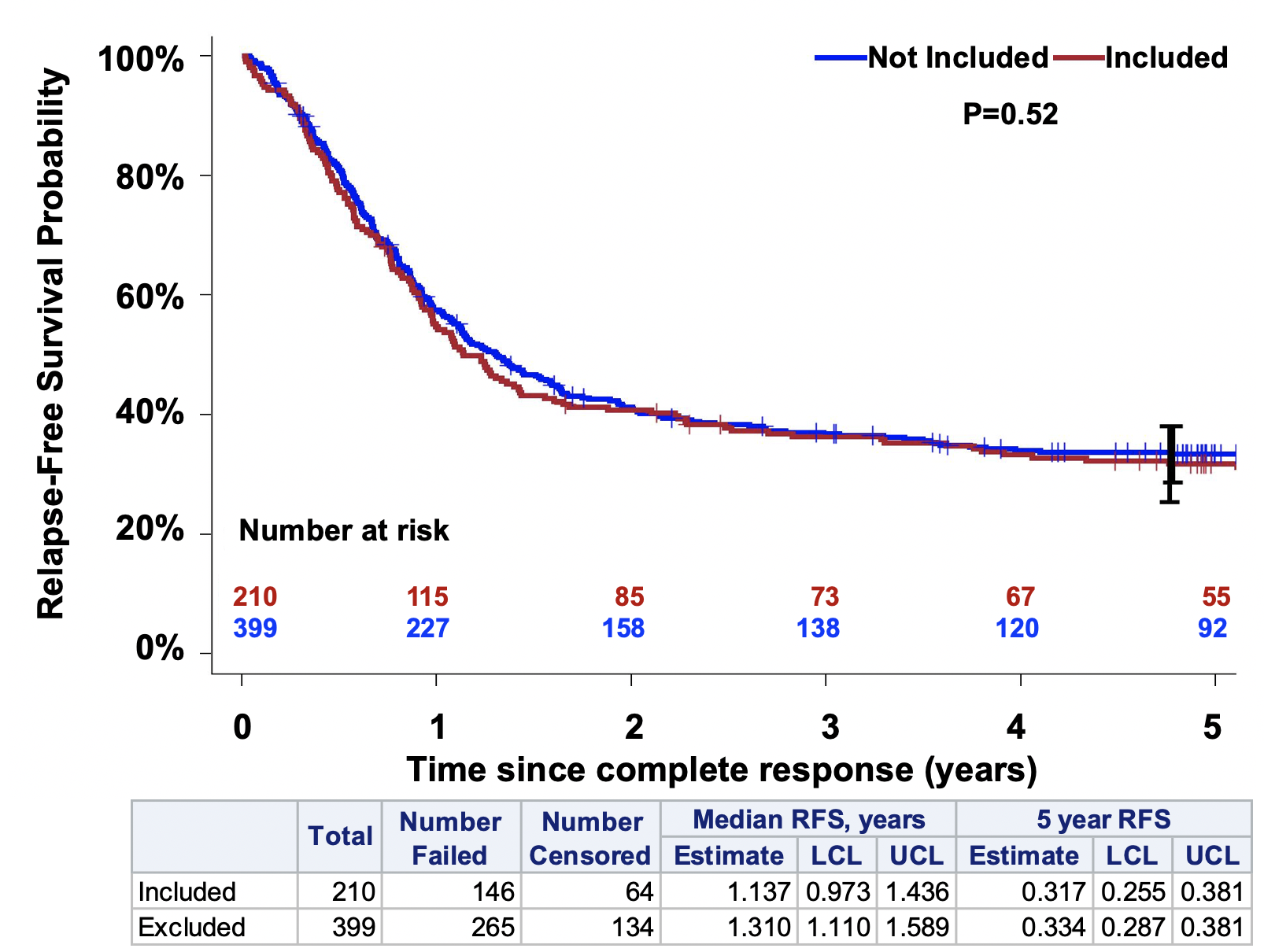


Fig. S1: Figure shows the probability (y-axis) for Relapse Free Survival (A) and Overall Survival (B) over the 5-year follow up period (x-axis) for SWOG patients that were included in this study (red line) and not included (blue line).

Table S4: Characteristics of SWOG patients in the discovery and validation cohorts

| **Characteristic** | | **Total (N=351)** | | **Discovery (N=185)** | | **Validation (N=166)** | |  |
| --- | --- | --- | --- | --- | --- | --- | --- | --- |
|  |  | **No.** | | **No.** | | **No.** | | **P*** |
| Age (yrs) | median | 56.3 | | 57.3 | | 54.4 | | 0.0865 |
|  | Range | 18.5 -88.8 | | 18.7 -84.2 | | 18.5 -88.8 | |  |
|  | N Missing | 0 | | 0 | | 0 | |  |
| Marrow blasts (%) | median | 71 | | 70.5 | | 71 | | 0.9802 |
|  | Range | 7 -100 | | 17 -100 | | 7 -100 | |  |
|  | N Missing | 11 | | 5 | | 6 | |  |
| WBC (10^9/L) | median | 36.5 | | 31.1 | | 41.2 | | 0.0188 |
|  | Range | 0.7 -369.7 | | 0.9 -307.6 | | 0.7 -369.7 | |  |
|  | N Missing | 0 | | 0 | | 0 | |  |
| PB Blasts (%) | median | 41 | | 43 | | 39 | | 0.7742 |
|  | Range | 0 -99 | | 0 -99 | | 0 -99 | |  |
|  | N Missing | 10 | | 6 | | 4 | |  |
| PB Blasts (10^9/L) | median | 10.1 | | 9.5 | | 11 | | 0.3117 |
|  | Range | 0 -347.5 | | 0 -261.5 | | 0 -347.5 | |  |
|  | N Missing | 10 | | 6 | | 4 | |  |
| Neutrophils (%) | median | 8 | | 8 | | 8 | | 0.8844 |
|  | Range | 0 -96 | | 0 -62 | | 0 -96 | |  |
|  | N Missing | 4 | | 2 | | 2 | |  |
| ANC (10^9/L) | median | 2.1 | | 1.8 | | 2.2 | | 0.1672 |
|  | Range | 0 -106.9 | | 0 -34.4 | | 0 -106.9 | |  |
|  | N Missing | 4 | | 2 | | 2 | |  |
| Hg (g/dL) | median | 9.1 | | 9.2 | | 9 | | 0.3255 |
|  | Range | 4.4 -29.1 | | 4.4 -29.1 | | 4.9 -14.4 | |  |
|  | N Missing | 8 | | 4 | | 4 | |  |
| PLT (10^9/L) | median | 52.5 | | 54 | | 52 | | 0.7656 |
|  | Range | 6 -449 | | 9 -412 | | 6 -449 | |  |
|  | N Missing | 1 | | 1 | | 0 | |  |
| Viability by DAPI | median | 74.1 | | 67.1 | | 76.5 | | 0.0010 |
|  | Range | 5.2 -97.2 | | 5.2 -95.6 | | 6.6 -97.2 | |  |
|  | N Missing | 2 | | 2 | | 0 | |  |
| Lymphocytes (%) | median | 7.3 | | 7.1 | | 7.5 | | 0.1543 |
|  | Range | 0.1 -72.9 | | 0.4 -70.1 | | 0.1 -72.9 | |  |
|  | N Missing | 5 | | 5 | | 0 | |  |
| Registration year | median | 2005 | | 2005 | | 2005 | | 0.9949 |
|  | Range | 1992 -2009 | | 1992 -2009 | | 1992 -2009 | |  |
|  | N Missing | 0 | | 0 | | 0 | |  |
| **Characteristic** | | **No.** | **%** | **No.** | **%** | **No.** | **%** | **P*** |
| Sex | Female | 169 | 48% | 81 | 44% | 88 | 53% | 0.0841 |
|  | Male | 182 | 52% | 104 | 56% | 78 | 47% |  |
| Race | White | 291 | 83% | 158 | 85% | 133 | 80% | 0.4724 |
|  | Black | 37 | 11% | 17 | 9% | 20 | 12% |  |
|  | Asian | 11 | 3% | 6 | 3% | 5 | 3% |  |
|  | Am. Indian or Alaska Native | 2 | 1% | 0 | 0% | 2 | 1% |  |
|  | Unknown | 10 | 3% | 4 | 2% | 6 | 4% |  |
| Hispanic ethnicity | Yes | 14 | 4% | 6 | 3% | 8 | 5% | 0.6185 |
|  | No | 288 | 82% | 155 | 84% | 133 | 80% |  |
|  | Unknown | 49 | 14% | 24 | 13% | 25 | 15% |  |
| Secondary AML | No | 322 | 92% | 166 | 90% | 156 | 94% | 0.1491 |
|  | Yes | 29 | 8% | 19 | 10% | 10 | 6% |  |
| Cytogenetics | -5/5q-,-7/7q- | 22 | 6% | 14 | 8% | 8 | 5% | 0.0296 |
|  | CBF | 40 | 11% | 17 | 9% | 23 | 14% |  |
|  | Normal | 127 | 36% | 57 | 31% | 70 | 42% |  |
|  | Normal+Nonclonal | 7 | 2% | 7 | 4% | 0 | 0% |  |
|  | Other | 76 | 22% | 38 | 21% | 38 | 23% |  |
|  | Missing | 79 | 23% | 52 | 28% | 27 | 16% |  |
| FAB class | M0 | 22 | 6% | 11 | 6% | 11 | 7% | 0.4569 |
|  | M1 | 75 | 21% | 41 | 22% | 34 | 20% |  |
|  | M2 | 83 | 24% | 51 | 28% | 32 | 19% |  |
|  | M3 | 1 | 0% | 1 | 1% | 0 | 0% |  |
|  | M4/M4eos | 111 | 32% | 54 | 29% | 57 | 34% |  |
|  | M5 | 47 | 13% | 21 | 11% | 26 | 16% |  |
|  | M6 | 1 | 0% | 0 | 0% | 1 | 1% |  |
|  | M7 | 1 | 0% | 1 | 1% | 0 | 0% |  |
|  | Other | 4 | 1% | 2 | 1% | 2 | 1% |  |
|  | Missing | 6 | 2% | 3 | 2% | 3 | 2% |  |
| Performance status | 0 | 108 | 31% | 63 | 34% | 45 | 27% | 0.0662 |
|  | 1 | 166 | 47% | 89 | 48% | 77 | 46% |  |
|  | 2 | 48 | 14% | 17 | 9% | 31 | 19% |  |
|  | 3 | 28 | 8% | 15 | 8% | 13 | 8% |  |
|  | Missing | 1 | 0% | 1 | 1% | 0 | 0% |  |
| Clinical trial | S0106 | 206 | 59% | 99 | 54% | 107 | 64% | 0.0209 |
|  | S0112 | 23 | 7% | 15 | 8% | 8 | 5% |  |
|  | S9031 | 57 | 16% | 27 | 15% | 30 | 18% |  |
|  | S9333 | 65 | 19% | 44 | 24% | 21 | 13% |  |
| Immunophenotype | CD117 | 88 | 25% | 35 | 19% | 53 | 32% | 0.0006 |
|  | CD34 | 188 | 54% | 99 | 54% | 89 | 54% |  |
|  | Double Negative | 72 | 21% | 50 | 27% | 22 | 13% |  |
|  | Not Done | 1 | 0% | 1 | 1% | 0 | 0% |  |
|  | Inconclusive | 2 | 1% | 0 | 0% | 2 | 1% |  |
| Cytogenetic risk | Favorable | 42 | 12% | 19 | 10% | 23 | 14% | 0.0280 |
|  | Intermediate | 182 | 52% | 86 | 46% | 96 | 58% |  |
|  | Unfavorable | 51 | 15% | 30 | 16% | 21 | 13% |  |
|  | Unknown | 76 | 22% | 50 | 27% | 26 | 16% |  |
| Complete response | No | 141 | 40% | 79 | 43% | 62 | 37% | 0.3071 |
|  | Yes | 210 | 60% | 106 | 57% | 104 | 63% |  |

Abbreviations: WBC, white blood cell count; PB, peripheral blood; ANC, absolute neutrophil count; Hg, hemoglobin; PLT, platelet count; AML, acute myeloid leukaemia; FAB, French American British classification.

*****P-value from Wilcoxon rank-sum tests for continuous variables and chi-squared or Fisher’s exact tests for categorical variables, depending on the frequency distribution. Missing values were excluded from testing.

Fig. S2: Comparison of Survival Outcomes of SWOG patients in the discovery and validation cohorts.

**B. Overall Survival**


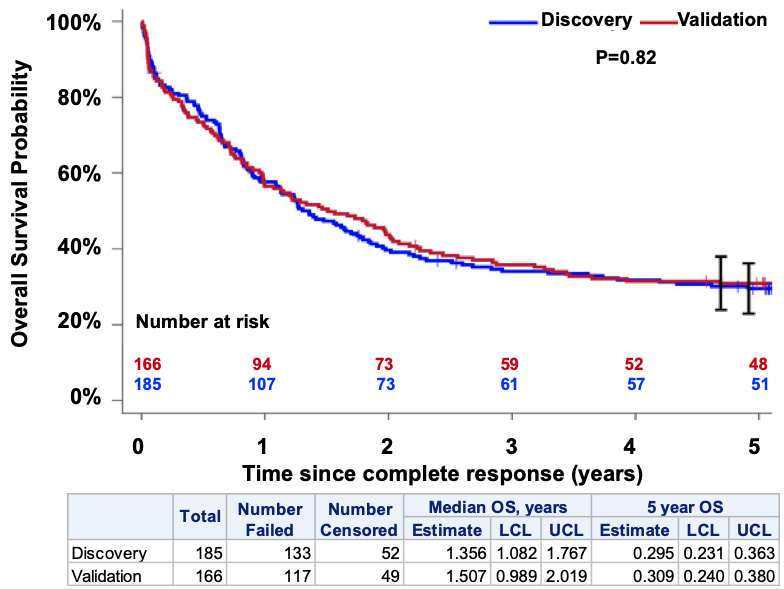


**A. Relapse Free Survival**


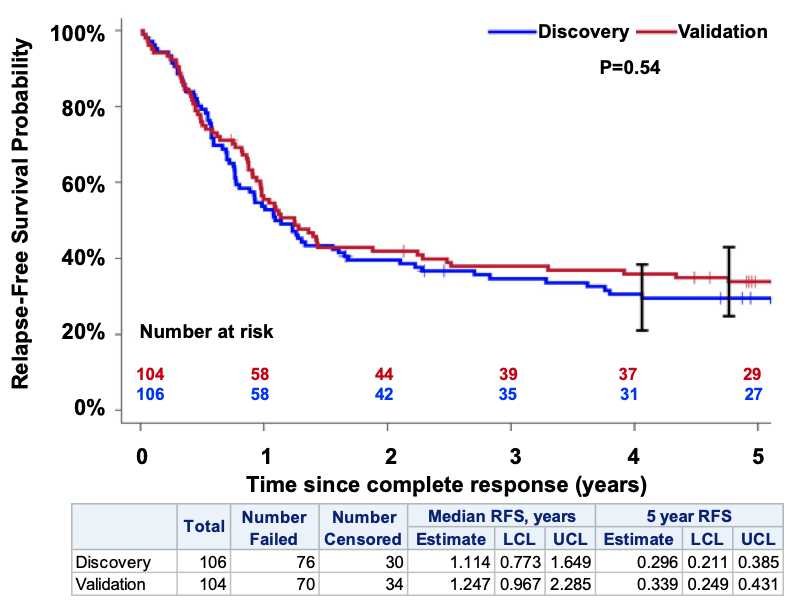


Fig. S2: Figure shows the probability (y-axis) for Relapse Free Survival (A) and Overall Survival (B) over the 5-year follow up period (x-axis) for SWOG patients in the discovery (blue line) and validation (red line) cohorts.

**Table S5: Mutation distribution in discovery and validation cohorts**

| **Characteristic** | | **Total**  **(N=351)** | | **Discovery (N=185)** | | **Validation (N=166)** | | **P*** |
| --- | --- | --- | --- | --- | --- | --- | --- | --- |
|  |  | **No.** | | **No.** | | **No.** | |  |
| *FLT3*-ITD AR (MNCs) | Median | 0.6 | | 0.6 | | 0.7 | | 0.0935 |
|  | Range | 0 -13.2 | | 0 -5.4 | | 0 -13.2 | |  |
|  | Negative | 240 | | 130 | | 110 | |  |
|  | Missing | 0 | | 0 | | 0 | |  |
| *FLT3*-ITD AR (VLBs) | Median | 0.8 | | 0.7 | | 0.8 | | 0.0646 |
|  | Range | 0 -20 | | 0 -20 | | 0.2 -20 | |  |
|  | Negative | 234 | | 123 | | 111 | |  |
|  | Missing | 6 | | 0 | | 6 | |  |
| **Characteristic** | | **No.** | **%** | **No.** | **%** | **No.** | **%** | **P*** |
| *ASXL1*  (all that agree) | Wild type | 314 | 89% | 159 | 86% | 155 | 93% | 0.0439 |
|  | Mutant | 35 | 10% | 24 | 13% | 11 | 7% |  |
|  | Missing | 2 | 1% | 2 | 1% | 0 | 0% |  |
| *FLT3*-ITD  (all that agree) | Wild type | 240 | 68% | 130 | 70% | 110 | 66% | 0.4225 |
|  | ITD | 109 | 31% | 54 | 29% | 55 | 33% |  |
|  | Missing | 2 | 1% | 1 | 1% | 1 | 1% |  |
| *NPM1* | Wild type | 225 | 64% | 123 | 66% | 102 | 61% | 0.2923 |
|  | Mutant | 125 | 36% | 61 | 33% | 64 | 39% |  |
|  | Missing | 1 | 0% | 1 | 1% | 0 | 0% |  |
| *RUNX1* | Wild type | 311 | 89% | 160 | 86% | 151 | 91% | 0.1875 |
|  | Mutant | 40 | 11% | 25 | 14% | 15 | 9% |  |
| *TP53* | Wild type | 325 | 93% | 167 | 90% | 158 | 95% | 0.0795 |
|  | Mutant | 26 | 7% | 18 | 10% | 8 | 5% |  |
| *CEBPA* | Wild type | 330 | 94% | 176 | 95% | 154 | 93% | 0.2462 |
|  | Mutant | 20 | 6% | 8 | 4% | 12 | 7% |  |
|  | Missing | 1 | 0% | 1 | 1% | 0 | 0% |  |

* P-value from Wilcoxon rank-sum tests for continuous variables and chi-squared tests for categorical variables. Missing values were excluded from testing.

**Fig. S3: Oncoprint, Mutation frequency**

Fig. S3: Mutation OncoPrint. Figure shows the mutation distribution for *NPM1*, *FLT3*-ITD, *RUNX1*, *ASXL1*, *TP53* and *CEBPA* (rows 3-8, green) in individual patients within the discovery (purple) and validation (orange) cohorts (top bar) stratified by the immunophenotype (IP: CD34, green; CD117, purple; or double negative, DN, orange, second bar). Missing data is identified by dark gray bars.

**Table S6: Expression fold change differences between MNCs and VLBs in discovery cohort**

| **Biomarker** | **MNCs** | | **VLBs** | | **P-value*** |
| --- | --- | --- | --- | --- | --- |
|  | **Mean** | **Range** | **Mean** | **Range** |  |
| *BAALC* | 18.252 | (0.003 - 260.816) | 29.237 | (0.0001 - 369.468) | <0.0001 |
| *CCNA1* | 57.403 | (0.001 - 1802.729) | 71.034 | (0.001 - 2153.210) | 0.0059 |
| *CEBPA* | 3.309 | (0.008 - 46.232) | 3.158 | (0.004 - 43.903) | 0.3553 |
| *ERG1* | 5.028 | (0 - 27.368) | 6.524 | (0 - 33.532) | <0.0001 |
| *EVI1* | 6.281 | (0.0003 - 333.139) | 7.022 | (0.0003 - 370.968) | 0.0007 |
| *FLT3* | 9.477 | (0.332 - 116.304) | 9.990 | (0.266 - 108.116) | 0.0243 |
| *GATA2* | 6.409 | (0.004 - 90.883) | 6.334 | (0.004 - 75.729) | 0.3544 |
| *IL3RA* | 3.927 | (0.029 - 49.363) | 4.220 | (0.0003 - 39.001) | 0.0693 |
| *JAG1* | 7.453 | (0.0004 - 64.092) | 7.348 | (0.0001 - 154.053) | 0.0739 |
| *KIT* | 4.565 | (0.0001 - 46.525) | 4.732 | (0.007 - 33.324) | 0.4152 |
| *MN1* | 21.133 | (0.0001 - 210.250) | 25.441 | (0.0002 - 296.055) | <0.0001 |
| *RUNX1* | 2.900 | (0.117 - 18.596) | 3.458 | (0.003 - 23.120) | 0.0012 |
| *WT1* | 0.118 | (0 - 1.077) | 0.144 | (0 - 1.027) | <0.0001 |
| *FLT3* (AR) | 0.729 | (0.04 - 5.36) | 1.420 | (0.03 - 20) | 0.0012 |

*P-value from Wilcoxon signed rank test for paired data. Mean and range are shown for paired samples only (patients with only MNCs or only VLBs not included).

**Table S7: Univariate Analyses Results, non-significant findings**

| **Variable** | | **Cell Population** | | | | | |
| --- | --- | --- | --- | --- | --- | --- | --- |
| **Biomarker** | **Value** | **MNCs** | | | **Blasts** | | |
| **Complete Response, CR** | | **N** | **OR (95% CI)** | **P** | **N** | **OR (95% CI)** | **P** |
| FLT3-ITD AR, NPM1+ | Continuous | 63 | 0.86 (0.66 - 1.13) | 0.28 | 60 | 0.93 (0.84 - 1.04) | 0.22 |
|  | IQR |  | 0.93 (0.82 - 1.06) |  |  | 0.96 (0.90 - 1.03) |  |
| BAALC | Continuous | 178 | 0.99 (0.98 - 1.00) | 0.079 | 162 | 1.00 (0.99 - 1.00) | 0.12 |
|  | IQR |  | 0.86 (0.72 - 1.02) |  |  | 0.87 (0.73 - 1.04) |  |
| CCNA1 | Continuous | 178 | 1.00 (1.00 - 1.00) | 0.47 | 162 | 1.00 (1.00 - 1.00) | 0.7 |
|  | IQR |  | 0.97 (0.89 - 1.06) |  |  | 0.98 (0.90 - 1.07) |  |
| CEBPA | Continuous | 178 | 0.98 (0.93 - 1.04) | 0.54 | 162 | 0.96 (0.89 - 1.03) | 0.25 |
|  | IQR |  | 0.95 (0.82 - 1.11) |  |  | 0.89 (0.72 - 1.09) |  |
| FLT3 | Continuous | 178 | 0.99 (0.97 - 1.02) | 0.58 | 162 | 0.99 (0.97 - 1.02) | 0.56 |
|  | IQR |  | 0.95 (0.78 - 1.15) |  |  | 0.95 (0.78 - 1.14) |  |
| GATA2 | Continuous | 178 | 0.97 (0.93 - 1.00) | 0.066 | 162 | 0.96 (0.92 - 1.00) | 0.052 |
|  | IQR |  | 0.80 (0.63 - 1.02) |  |  | 0.69 (0.47 - 1.00) |  |
| IL3RA | Continuous | 178 | 1.01 (0.95 - 1.07) | 0.82 | 162 | 0.99 (0.94 - 1.06) | 0.87 |
|  | IQR |  | 1.03 (0.82 - 1.29) |  |  | 0.98 (0.79 - 1.22) |  |
| JAG1 | Continuous | 178 | 0.99 (0.96 - 1.01) | 0.3 | 162 | 1.00 (0.98 - 1.02) | 0.89 |
|  | IQR |  | 0.90 (0.74 - 1.10) |  |  | 0.99 (0.85 - 1.15) |  |
| RUNX1 | Continuous | 178 | 0.93 (0.84 - 1.05) | 0.23 | 162 | 0.92 (0.83 - 1.01) | 0.074 |
|  | IQR |  | 0.84 (0.64 - 1.12) |  |  | 0.71 (0.49 - 1.03) |  |
| WT1 | Continuous | 178 | 0.61 (0.13 - 2.90) | 0.53 | 162 | 0.51 (0.09 - 2.81) | 0.44 |
|  | IQR |  | 0.93 (0.73 - 1.17) |  |  | 0.88 (0.63 - 1.22) |  |
| **Overall Survival, OS** | | **N** | **HR (95% CI)** | **P** | **N** | **HR (95% CI)** | **P** |
| FLT3-ITD AR, NPM1+ | Continuous | 63 | 1.13 (1.00 - 1.26) | 0.043 | 60 | 1.04 (0.98 - 1.11) | 0.16 |
|  | IQR |  | 1.06 (1.00 - 1.12) |  |  | 1.03 (0.99 - 1.06) |  |
| BAALC | Continuous | 178 | 1.00 (1.00 - 1.01) | 0.15 | 162 | 1.00 (1.00 - 1.01) | 0.12 |
|  | IQR |  | 1.06 (0.98 - 1.14) |  |  | 1.07 (0.98 - 1.16) |  |
| CEBPA | Continuous | 178 | 1.02 (0.99 - 1.05) | 0.24 | 162 | 1.03 (0.99 - 1.07) | 0.12 |
|  | IQR |  | 1.05 (0.97 - 1.14) |  |  | 1.09 (0.98 - 1.21) |  |
| GATA2 | Continuous | 178 | 1.00 (0.98 - 1.02) | 0.99 | 162 | 1.01 (1.00 - 1.03) | 0.16 |
|  | IQR |  | 1.00 (0.89 - 1.12) |  |  | 1.10 (0.96 - 1.27) |  |
| JAG1 | Continuous | 178 | 1.01 (0.99 - 1.02) | 0.22 | 162 | 1.00 (0.99 - 1.01) | 0.68 |
|  | IQR |  | 1.07 (0.96 - 1.20) |  |  | 1.02 (0.94 - 1.09) |  |
| KIT | Continuous | 178 | 1.01 (0.99 - 1.04) | 0.42 | 162 | 1.00 (0.97 - 1.03) | 0.82 |
|  | IQR |  | 1.04 (0.94 - 1.16) |  |  | 1.02 (0.88 - 1.17) |  |
| MN1 | Continuous | 178 | 1.00 (1.00 - 1.01) | 0.72 | 162 | 1.00 (1.00 - 1.00) | 0.78 |
|  | IQR |  | 1.02 (0.93 - 1.12) |  |  | 1.01 (0.92 - 1.12) |  |
| RUNX1 | Continuous | 178 | 1.04 (0.98 - 1.10) | 0.2 | 162 | 1.04 (1.00 - 1.09) | 0.067 |
|  | IQR |  | 1.10 (0.95 - 1.28) |  |  | 1.18 (0.99 - 1.41) |  |
| WT1 | Continuous | 178 | 0.75 (0.27 - 2.08) | 0.58 | 162 | 0.78 (0.28 - 2.14) | 0.62 |
|  | IQR |  | 0.96 (0.82 - 1.12) |  |  | 0.95 (0.78 - 1.16) |  |
| **Relapse-Free Survival, RFS** | | **N** | **HR (95% CI)** | **P** | **N** | **HR (95% CI)** | **P** |
| FLT3-ITD AR, NPM1+ | Continuous | 39 | 1.05 (0.87 - 1.28) | 0.59 | 36 | 1.04 (0.96 - 1.14) | 0.34 |
|  | IQR |  | 1.03 (0.94 - 1.12) |  |  | 1.03 (0.97 - 1.08) |  |
| BAALC | Continuous | 101 | 1.00 (0.99 - 1.01) | 0.9 | 91 | 1.00 (1.00 - 1.01) | 0.4 |
|  | IQR |  | 0.99 (0.81 - 1.20) |  |  | 1.07 (0.92 - 1.25) |  |
| CEBPA | Continuous | 101 | 1.03 (0.98 - 1.08) | 0.29 | 91 | 1.01 (0.94 - 1.09) | 0.7 |
|  | IQR |  | 1.08 (0.94 - 1.24) |  |  | 1.04 (0.84 - 1.29) |  |
| ERG1 | Continuous | 101 | 1.01 (0.97 - 1.06) | 0.56 | 91 | 1.03 (1.00 - 1.07) | 0.081 |
|  | IQR |  | 1.07 (0.86 - 1.33) |  |  | 1.29 (0.97 - 1.72) |  |
| FLT3 | Continuous | 101 | 1.01 (0.99 - 1.03) | 0.28 | 91 | 1.01 (0.99 - 1.03) | 0.29 |
|  | IQR |  | 1.08 (0.94 - 1.25) |  |  | 1.08 (0.94 - 1.24) |  |
| GATA2 | Continuous | 101 | 0.98 (0.95 - 1.02) | 0.37 | 91 | 1.01 (0.97 - 1.05) | 0.63 |
|  | IQR |  | 0.89 (0.70 - 1.14) |  |  | 1.09 (0.78 - 1.51) |  |
| JAG1 | Continuous | 101 | 1.01 (0.99 - 1.03) | 0.48 | 91 | 1.00 (0.99 - 1.01) | 0.6 |
|  | IQR |  | 1.06 (0.90 - 1.24) |  |  | 1.02 (0.94 - 1.11) |  |
| KIT | Continuous | 101 | 1.01 (0.97 - 1.04) | 0.71 | 91 | 1.01 (0.96 - 1.06) | 0.7 |
|  | IQR |  | 1.03 (0.88 - 1.20) |  |  | 1.05 (0.83 - 1.31) |  |
| MN1 | Continuous | 101 | 1.00 (0.99 - 1.01) | 0.89 | 91 | 1.00 (0.99 - 1.01) | 0.7 |
|  | IQR |  | 1.01 (0.87 - 1.17) |  |  | 1.04 (0.86 - 1.26) |  |
| RUNX1 | Continuous | 101 | 1.02 (0.94 - 1.10) | 0.66 | 91 | 1.04 (0.97 - 1.12) | 0.29 |
|  | IQR |  | 1.05 (0.86 - 1.28) |  |  | 1.16 (0.88 - 1.54) |  |
| WT1 | Continuous | 101 | 0.74 (0.18 - 3.12) | 0.68 | 91 | 1.24 (0.31 - 5.03) | 0.76 |
|  | IQR |  | 0.96 (0.77 - 1.19) |  |  | 1.04 (0.80 - 1.37) |  |

Transcript expression fold changes were analyzed both as unadjusted variables (Cont.) and adjusted (divided) by the interquartile range (IQR) of the corresponding expression variable in the discovery data.

**Models Details**

These details contain R code and output for the final models produced by the algorithm described in the main paper. Models were built separately for each outcome (OS, RFS, CR) and for each type of cells (MNCs and blasts). Examples are given to illustrate how different values of each covariate impact a hypothetical patient’s probability of achieving CR or risk of relapse or death.

**Overall Survival**

**MNCs:**

> coxph(Surv(SURTIM, SURIND) ~ AGE + ELN + ERG1 + EVI1 + JAG1 + ELN:JAG1, data=datat0)

Call:

coxph(formula = Surv(SURTIM, SURIND) ~ AGE + ELN + ERG1 +

EVI1 + JAG1 + ELN:JAG1, data = datat0)

coef exp(coef) se(coef) z p

AGE 0.057655 1.059349 0.007834 7.360 1.84e-13

ELNAdverse 0.450146 1.568541 0.376064 1.197 0.2313

ELNFavorable 0.118981 1.126349 0.405543 0.293 0.7692

ELNUnknown 0.750523 2.118108 0.400016 1.876 0.0606

ERG1 0.029921 1.030373 0.018084 1.655 0.0980

EVI1 0.005175 1.005188 0.002342 2.210 0.0271

JAG1 0.089656 1.093798 0.041792 2.145 0.0319

ELNAdverse:JAG1 -0.055747 0.945778 0.043982 -1.268 0.2050

ELNFavorable:JAG1-0.095132 0.909253 0.045045 -2.112 0.0347

ELNUnknown:JAG1 -0.093796 0.910468 0.043593 -2.152 0.0314

Likelihood ratio test=92.16 on 10 df, p=1.993e-15

n= 178, number of events= 128

The four “risk groups” defined by the model in the discovery cohort are as follows:

1^st^ quartile (lowest risk): ≤ 3.00

2^nd^ quartile: (3.00, 4.06]

3^rd^ quartile: (4.06, 4.65]

4^th^ quartile (highest risk): > 4.65

For example, a patient aged 60 years with Adverse ELN risk, ERG1 = 15.8, EVI1 = 0.18, JAG1 = 0:

0.0577*(60) + 0.4501*(1) + 0.1190*(0) + 0.7505*(0)+ 0.0299*(15.8)+ 0.0052*(0.18) + 0.0897*(0) - 0.0557*(1*0) -0.0951*(0*0) -0.0938* (0*0) = 4.39

This patient would fall into the 3^rd^ risk group.

Another patient, aged 40 years with Intermediate ELN risk, ERG1 = 0.13, EVI1 = 0, JAG1 = 0.07:

0.0577*(40) + 0.4501*(0) + 0.1190*(0) + 0.7505*(0)+ 0.0299*(0.13)+ 0.0052*(0) + 0.0897*(0.07) - 0.0557*(0*0.07) -0.0951*(0*0.07) -0.0938* (1*0.07) = 2.31

This patient would fall into the 1^st^ risk group.

**Blasts:**

> coxph(Surv(SURTIM, SURIND) ~ AGE + ELN_B1 + EVI1_B1 + ERG1_B1 + CCNA1_B1, data=datab1)

Call:

coxph(formula = Surv(SURTIM, SURIND) ~ AGE + ELN_B1 + EVI1_B1 +

ERG1_B1 + CCNA1_B1, data = datab1)

coef exp(coef) se(coef) z p

AGE 0.0510072 1.0523304 0.0079516 6.415 1.41e-10

ELN_B1Adverse 0.0451177 1.0461510 0.2876724 0.157 0.8754

ELN_B1Favorable -0.5550882 0.5740216 0.3233422 -1.717 0.0860

ELN_B1Unknown 0.2448697 1.2774548 0.2957343 0.828 0.4077

EVI1_B1 0.0052446 1.0052584 0.0024434 2.146 0.0318

ERG1_B1 0.0292303 1.0296617 0.0143178 2.042 0.0412

CCNA1_B1 0.0008558 1.0008561 0.0004952 1.728 0.0840

Likelihood ratio test=78.05 on 7 df, p=3.44e-14

n= 162, number of events= 120

The four “risk groups” defined by the model in the discovery cohort are as follows:

1^st^ quartile (lowest risk): ≤ 2.27

2^nd^ quartile: (2.27, 3.22]

3^rd^ quartile: (3.22, 3.85]

4^th^ quartile (highest risk): > 3.85

For example, a patient aged 44 years with Adverse ELN risk, EVI1 = 0, ERG1 = 7.2, CCNA1 = 7.6:

0.0510*(44) + 0.0451*(1) - 0.5551*(0) + 0.2449*(0) + 0.0052*(0) + 0.0292*(7.2) + 0.0009*(7.6) = 2.51

This patient would fall into the 2^nd^ risk group.

Another patient, aged 58 years with Favorable ELN risk, , EVI1 = 0.062, ERG1 = 5.10, CCNA1 = 50.91:

0.0510*(58) + 0.0451*(0) - 0.5551*(1) + 0.2449*(0) + 0.0052*(0.062) + 0.0292*(5.10) + 0.0009*(50.91) = 2.60

This patient would also fall into the 2^nd^ risk group.

**Relapse-Free Survival**

**MNCs:**

> coxph(Surv(RFSTIM, RFSIND) ~ AGE + ELN_T0 + EVI1_T0 + CCNA1_T0, data=datat0)

Call:

coxph(formula = Surv(RFSTIM, RFSIND) ~ AGE + ELN_T0 + EVI1_T0 +

CCNA1_T0, data = datat0)

Coef exp(coef) se(coef) z p

AGE 0.022790 1.023052 0.008999 2.533 0.011323

ELN_T0Adverse 1.018995 2.770410 0.272969 3.733 0.000189

EVI1_T0 0.015929 1.016056 0.005336 2.985 0.002836

CCNA1_T0 0.003510 1.003516 0.001525 2.301 0.021383

Likelihood ratio test=35.75 on 4 df, p=3.251e-07

n= 101, number of events= 73

The four “risk groups” defined by the model in the discovery cohort are as follows:

1^st^ quartile (lowest risk): ≤ 1.01

2^nd^ quartile: (1.01, 1.43]

3^rd^ quartile: (1.43, 2.18]

4^th^ quartile (highest risk): > 2.18

For example, a patient aged 72 years with Adverse ELN risk, EVI1 = 87.14, CCNA1 = 10.01:

0.0228*(72) + 1.0190*(1) + 0.0159*(87.14) + 0.0035*(10.01) = 4.08

This patient would fall into the 4^th^ risk group.

Another patient, aged 36 years with Unknown ELN risk, EVI1 = 0, CCNA1 = 11.19:

0.0228*(36) + 1.0190*(0) + 0.0159*(0) + 0.0035*(11.19) = 0.86

This patient would fall into the 1^st^ risk group.

**Blasts:**

> coxph(Surv(RFSTIM, RFSIND) ~ AGE + ELN_B1 + EVI1_B1 + CCNA1_B1, data=datab1)

Call:

coxph(formula = Surv(RFSTIM, RFSIND) ~ AGE + ELN_B1 + EVI1_B1 +

CCNA1_B1, data = datab1)

coef exp(coef) se(coef) z p

AGE 0.0188577 1.0190366 0.0097114 1.942 0.052159

ELN_B1Adverse 1.0260804 2.7901082 0.2841832 3.611 0.000305

EVI1_B1 0.0162788 1.0164121 0.0062708 2.596 0.009432

CCNA1_B1 0.0017304 1.0017319 0.0009977 1.734 0.082855

Likelihood ratio test=29.59 on 4 df, p=5.934e-06

n= 91, number of events= 68

The four “risk groups” defined by the model in the discovery cohort are as follows:

1^st^ quartile (lowest risk): ≤ 0.84

2^nd^ quartile: (0.84, 1.19]

3^rd^ quartile: (1.19, 2.04]

4^th^ quartile (highest risk): > 2.04

For example, a patient aged 54 years with Intermediate ELN risk, EVI1 = 0, CCNA1 = 5.83:

0.0189*(54) + 1.0260*(0) + 0.0163*(0) + 0.0017*(5.83) = 1.03

This patient would fall into the 2^nd^ risk group.

Another patient, aged 56 years with Adverse ELN risk, EVI1 = 0.04, CCNA1 = 1.14:

0.0189*(56) + 1.0260*(1) + 0.0163*(0.04) + 0.0017*(1.14) = 2.09

This patient would fall into the 4^th^ risk group.

**Complete Response**

**MNCs:**

> summary(glm(CR ~ AGE + PS + GATA2_T0 + BAALC_T0, data=datat0, family=binomial(link="logit")))

Call:

glm(formula = CR ~ AGE + PS + GATA2_T0 + BAALC_T0, family = binomial(link = "logit"), data = datat0)

Deviance Residuals:

Min 1Q Median 3Q Max

-1.8710 -1.1107 0.6543 0.9587 1.7013

Coefficients:

Estimate Std. Error z value Pr(>|z|)

(Intercept) 3.038788 0.699580 4.344 1.4e-05 ***

AGE -0.038915 0.011289 -3.447 0.000566 ***

PS2 - 3 -0.909079 0.431626 -2.106 0.035189 *

GATA2_T0 -0.038585 0.020813 -1.854 0.063759 .

BAALC_T0 -0.010302 0.005538 -1.860 0.062862 .

---

Signif. codes: 0 ‘***’ 0.001 ‘**’ 0.01 ‘*’ 0.05 ‘.’ 0.1 ‘ ’ 1

(Dispersion parameter for binomial family taken to be 1)

Null deviance: 241.83 on 176 degrees of freedom

Residual deviance: 215.36 on 172 degrees of freedom

AIC: 225.36

Number of Fisher Scoring iterations: 4

The four “risk groups” defined by the model in the discovery cohort are as follows:

1^st^ quartile (lowest probability of CR): ≤ -0.20

2^nd^ quartile: (-0.20, 0.35]

3^rd^ quartile: (0.35, 0.99]

4^th^ quartile (highest probability of CR): > 0.99

For example, a patient aged 61 years with PS = 2, GATA2 = 0.664, BAALC = 94.46:

3.0388 – 0.0389*(61) – 0.9091*(1) – 0.0386*(0.664) – 0.0103*(94.46) = -1.24

This patient would fall into the 1^st^ risk group, with a low probability of achieving a CR.

Another patient, aged 48 years with PS = 1, GATA2 = 2.05, BAALC = 2.22:

3.0388 – 0.0389*(48) – 0.9091*(0) – 0.0386*(2.05) – 0.0103*(2.22) = 1.07

This patient would fall into the 4^th^ risk group, with a high probability of achieving a CR.

**Blasts:**

> summary(glm(CR ~ AGE + PS + MN1_B1 + GATA2_B1, data=datab1, family=binomial(link="logit")))

Call:

glm(formula = CR ~ AGE + PS + MN1_B1 + GATA2_B1, family = binomial(link = "logit"), data = datab1)

Deviance Residuals:

Min 1Q Median 3Q Max

-1.9122 -1.0803 0.6128 0.9041 1.9013

Coefficients:

Estimate Std. Error z value Pr(>|z|)

(Intercept) 3.119632 0.754104 4.137 3.52e-05 ***

AGE -0.037653 0.012024 -3.131 0.00174 **

PS2 - 3 -1.050215 0.466547 -2.251 0.02438 *

MN1_B1 -0.011590 0.004687 -2.473 0.01340 *

GATA2_B1 -0.042702 0.023449 -1.821 0.06860 .

---

Signif. codes: 0 ‘***’ 0.001 ‘**’ 0.01 ‘*’ 0.05 ‘.’ 0.1 ‘ ’ 1

(Dispersion parameter for binomial family taken to be 1)

Null deviance: 220.45 on 160 degrees of freedom

Residual deviance: 191.35 on 156 degrees of freedom

AIC: 201.35

Number of Fisher Scoring iterations: 4

The four “risk groups” defined by the model in the discovery cohort are as follows:

1^st^ quartile (lowest risk): ≤ -0.29

2^nd^ quartile: (-0.29, 0.40]

3^rd^ quartile: (0.40, 0.98]

4^th^ quartile (highest risk): > 0.98

For example, a patient aged 69 years with PS = 0, MN1 = 3.28, GATA2 = 4.62:

3.1196 – 0.0377*(69) – 1.0502*(0) – 0.0116*(3.28) – 0.0427*(4.62) = 0.028

This patient would fall into the 2^nd^ risk group.

Another patient, aged 43 years with PS = 1, MN1 = 0, GATA2 = 1.21:

3.1196 – 0.0377*(43) – 1.0502*(0) – 0.0116*(0) – 0.0427*(1.21) = 1.45

This patient would fall into the 4^th^ risk group.

| **AGE, PS and multivariable models for CR, OS and RFS (for comparison)** | | | | | | | |
| --- | --- | --- | --- | --- | --- | --- | --- |
|  |  |  | |  | |  | |
| **Model** | | | **MNCs** | | **VLBs** | | |
| **Complete Response (CR, AUC)** | | | **Discovery** | **Validation** | **Discovery** | | **Validation** |
| ELN_2017_ | | | 0.66 | 0.73 | 0.66 | | 0.67 |
| AGE | | | 0.66 | 0.60 | 0.65 | | 0.62 |
| PS | | | 0.57 | 0.58 | 0.58 | | 0.59 |
| AGE+ELN_2017_ | | | 0.71 | 0.72 | 0.71 | | 0.72 |
| ELN+PS | | | 0.70 | 0.73 | 0.70 | | 0.73 |
| AGE+PS | | | 0.68 | 0.64 | 0.69 | | 0.65 |
| AGE+ELN_2017_+PS | | | 0.72 | 0.73 | 0.73 | | 0.73 |
| **Overall Survival (OS, C Statistic)** | | | **Discovery** | **Validation** | **Discovery** | | **Validation** |
| ELN_2017_ | | | 0.60 | 0.68 | 0.59 | | 0.68 |
| AGE | | | 0.71 | 0.65 | 0.69 | | 0.65 |
| PS | | | 0.54 | 0.57 | 0.54 | | 0.57 |
| AGE+ELN_2017_ | | | 0.72 | 0.70 | 0.71 | | 0.71 |
| ELN+PS | | | 0.62 | 0.71 | 0.63 | | 0.71 |
| AGE+PS | | | 0.71 | 0.66 | 0.69 | | 0.66 |
| AGE+ELN_2017_+PS | | | 0.72 | 0.70 | 0.71 | | 0.71 |
| **Relapse-Free Survival (RFS, C Statistic)** | | | **Discovery** | **Validation** | **Discovery** | | **Validation** |
| ELN_2017_^⍒^ | | | 0.6 | 0.54 | 0.61 | | 0.54 |
| AGE | | | 0.64 | 0.62 | 0.63 | | 0.61 |
| PS | | | 0.5 | 0.57 | 0.50 | | 0.57 |
| AGE + ELN_2017_^⍒^ | | | 0.67 | 0.63 | 0.66 | | 0.62 |
| ELN_2017_^⍒^+PS | | | 0.61 | 0.57 | 0.61 | | 0.57 |
| AGE+PS | | | 0.64 | 0.63 | 0.64 | | 0.63 |
| AGE+ELN_2017_^⍒^+PS | | | 0.67 | 0.68 | 0.67 | | 0.67 |

⍒Due to small sample size, ELN_2017_ in the RFS models was categorized into adverse vs. not adverse (including intermediate, favorable, and unknown).

**Table S8: ELN_2017_ and ELN_2017 MOD_ risk assignments in MNCs and VLBs**

| **MNCs** | **ELN_2017_** | | | |  |
| --- | --- | --- | --- | --- | --- |
| **ELN_2017 MOD_** | **Adverse** | **Intermediate** | **Favorable** | **Unknown** | **Total** |
| **Adverse** | 66 | 0 | 2 | 0 | 68 |
| **Intermediate** | 29 | 64 | 15 | 0 | 108 |
| **Favorable** | 0 | 0 | 99 | 0 | 99 |
| **Unknown** | 0 | 0 | 0 | 76 | 76 |
| **Total** | 95 | 64 | 116 | 76 | 351 |

| **VLBs** | **ELN_2017_** | | | |  |
| --- | --- | --- | --- | --- | --- |
| **ELN_2017 MOD_** | **Adverse** | **Intermediate** | **Favorable** | **Unknown** | **Total** |
| **Adverse** | 68 | 0 | 2 | 0 | 70 |
| **Intermediate** | 26 | 60 | 15 | 0 | 101 |
| **Favorable** | 0 | 0 | 94 | 0 | 94 |
| **Unknown** | 1 | 0 | 0 | 85 | 86 |
| **Total** | 95 | 60 | 111 | 85 | 351 |
